# Green Synthesis of Oxoquinoline-1(2H)-Carboxamide as Antiproliferative and Antioxidant Agents: An Experimental and In-Silico Approach to High Altitude Related Disorders

**DOI:** 10.3390/molecules27010309

**Published:** 2022-01-04

**Authors:** Amena Ali, Abuzer Ali, Musarrat Husain Warsi, Mohammad Akhlaquer Rahman, Mohamed Jawed Ahsan, Faizul Azam

**Affiliations:** 1 High Altitude Research Center, Taif University, P.O. Box 11099, Taif 21944, Saudi Arabia; 2 Department of Pharmaceutical Chemistry, College of Pharmacy, Taif University, P.O. Box 11099, Taif 21944, Saudi Arabia; 3 Department of Pharmacognosy, College of Pharmacy, Taif University, P.O. Box 11099, Taif 21944, Saudi Arabia; abuali@tu.edu.sa; 4 Department of Pharmaceutics and Industrial Pharmacy, College of Pharmacy, Taif University, P.O. Box 11099, Taif 21944, Saudi Arabia; mvarsi@tu.edu.sa (M.H.W.); mrahman@tu.edu.sa (M.A.R.); 5 Department of Pharmaceutical Chemistry, Maharishi Arvind College of Pharmacy, Jaipur 302 039, Rajasthan, India; jawedpharma@gmail.com; 6 Department of Pharmaceutical Chemistry & Pharmacognosy, Unaizah College of Pharmacy, Qassim University, Uniazah 51911, Saudi Arabia; f.azam@qu.edu.sa

**Keywords:** antioxidant, anticancer, carbonic anhydrase (CA), epidermal growth factor receptor (EGFR), green synthesis, high-altitude, oxidative stress, oxoquinolines

## Abstract

At high altitudes, drops in oxygen concentration result in the creation of reactive oxygen and nitrogen species (RONS), which cause a variety of health concerns. We addressed these health concerns and reported the synthesis, characterization, and biological activities of a series of 10 oxoquinolines. *N*-Aryl-7-hydroxy-4-methyl-2-oxoquinoline-1(2*H*)carboxamides (**5a**–**j**) were accessed in two steps under ultrasonicated irradiation, as per the reported method. The anticancer activity was tested at 10 µM against a total of 5 dozen cancer cell lines obtained from nine distinct panels, as per the National Cancer Institute (NCI US) protocol. The compounds **5a** (TK-10 (renal cancer); %GI = 82.90) and **5j** (CCRF-CEM (Leukemia); %GI = 58.61) showed the most promising anticancer activity. Compound **5a** also demonstrated promising DPPH free radical scavenging activity with an IC_50_ value of 14.16 ± 0.42 µM. The epidermal growth factor receptor (EGFR) and carbonic anhydrase (CA), two prospective cancer inhibitor targets, were used in the molecular docking studies. Molecular docking studies of ligand **5a** (docking score = −8.839) against the active site of EGFR revealed two H-bond interactions with the residues Asp855 and Thr854, whereas ligand **5a** (docking = −5.337) interacted with three H-bond with the residues Gln92, Gln67, and Thr200 against the active site CA. The reported compounds exhibited significant anticancer and antioxidant activities, as well as displayed significant inhibition against cancer targets, EGFR and CA, in the molecular docking studies. The current discovery may aid in the development of novel compounds for the treatment of cancer and oxidative stress, and other high altitude-related disorders.

## 1. Introduction

At high altitude, the concentration of oxygen decreases, resulting in the formation of reactive oxygen and nitrogen species (RONS) [1]. High altitude RONS production causes lipid, protein, and DNA damage, as well as weakening of the enzymatic and non-enzymatic antioxidant systems [2]. Excessive ROS production in particular has been linked to the development and augmentation of cardiovascular, pulmonary, neurodegenerative, and metabolic disorders, as well as cancer [3,4,5,6,7]. Furthermore, several ailments, including cancer, Alzheimer’s, and diabetes, as well as motion sickness, may occur as a result of high-altitude oxidative stress [8,9]. Aerobic respiration, apoptosis, cell growth and survival, nucleic acid synthesis, and oxidative stress are all controlled by mitochondrial signaling. Mitochondrial malfunction and the resulting mishandling of the reactive oxygen species (ROS) cascade have been linked to cancer progression [10,11]. Cancer, on the other hand, is one of the terrifying diseases that frighten scientists and researchers all over the world. In 2018, 18.1 million individuals were diagnosed with cancer, and 9.6 million died as a result of it; these numbers are expected to nearly double by 2040 [12]. Oxidative stress can potentially cause malignant cell growth [13].

The term “Green Chemistry” was coined in 1991 to describe the removal or reduction of hazardous compounds with the purpose of limiting chemical exposure to individuals and the environment [14]. As our globe faces the environmental concerns of the twenty-first century, the demand for green chemistry for the synthesis of compounds with therapeutic relevance is increasing. Green chemistry has been used for advanced technologies to minimize hazardous, unwanted waste, and environmental effects. Because of the advantages of green chemistry, such as reduced waste and cost, not only pharmaceutical companies but also other chemical industries, have begun to take steps toward it [15]. In today’s world, synthetic chemists in academia and industry are continually challenged to think of more environmentally friendly ways to generate the desired target molecules [16]. Ultrasound-mediated synthesis is a solution-based green synthesis, which offers a high conversion rate in a short amount of time. The ultrasound technique increased the reaction rate even under milder conditions as compared to traditional heating methods; it is also an effective energy-saving strategy [14,17,18,19]. Ultrasound causes acoustic cavitation during chemical reactions. High pressure (18,000 atomic pressures) and temperature (2000–5000 K) are generated by acoustic cavitation, which influence chemical transformations [20,21]. The use of ultrasonication in synthesis has grown in popularity in recent years [22,23,24,25,26].

Solvents have attracted a lot of attention in the context of green chemistry since they are a major source of contamination in organic chemical processes. As a result of the increasing environmental contamination caused by the exponential usage of volatile and poisonous organic solvents in chemical industries, chemists have been pushed to focus on alternative green solvents [27]. Solvent-free synthesis can help to reduce the dangers of volatile and poisonous organic solvents, although this is not a solution. Running a reaction in a solvent, on the other hand, is often desirable to enable mass and heat transfer. Furthermore, the correct solvent selection affects reaction rates, selectivity, and the location of chemical equilibria [28]. After all, water has numerous advantages because it is a low-cost, readily available, non-toxic, and non-flammable solvent that is appealing from both an economic and an environmental aspect [28]. Using water as a solvent, several research groups successfully synthesized quinoline, a bicyclic heterocycle [23,29,30,31]. We discussed the ultrasonic synthesis of quinolines in green solvent water, as well as their biological activity, in the current study.

**Figure 1:**
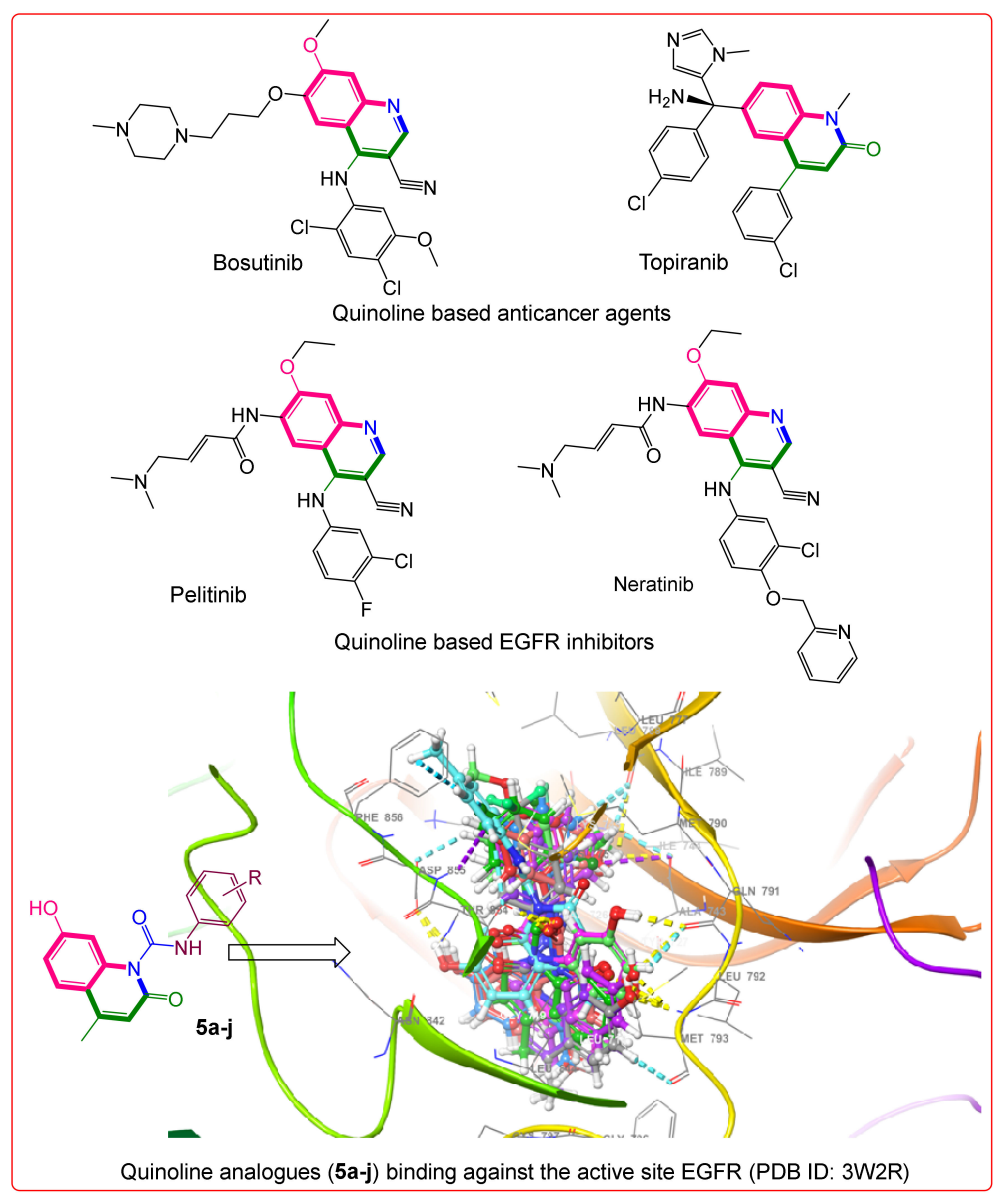
Quinoline-based anticancer agents and EGFR inhibitors and ligands (**5a**–**j**).

Quinoline is an important scaffold found in many anticancer drugs, including bosutinib, topiranib, pelitinib, neratinib, and others [32,33,34]. Some of the quinolines based anticancer agents and EGFR inhibitors and the target ligands (**5a**–**j**) are shown in Figure 1. The molecular docking scores of the title compounds (**5a**–**j**) were found to be greater than −7.944 Kcal/mol, indicating their efficient binding against the active site of epidermal growth factor receptor (EGFR), one of the most appealing targets for anticancer agents. Three types of interactions (H-bond, π-π-Stacking and Halogen bond) were observed in molecular docking simulation. Some of the ligands showed H-bond interaction of phenolic function with the residue Met793. Many biomarkers are triggered as a result of oxidative stress, which induces inflammation and the transformation of normal cells into malignant cells. Some of the target compounds with promising anticancer activity were also evaluated for antioxidant activity. Similarly, the carbonic anhydrase (CA) isozyme IX in some of the cancer cases promotes the proliferation and metastasis of tumors; it has also been observed to be overexpressed in many malignancies due to hypoxia [35,36]. As a result, CA could also be a promising anticancer treatment strategy. Some of the oxoquinolines bind to the CA very efficiently and display three sorts of interactions (H-bond, π-π-stacking and halogen bond).

## 2. Results

### 2.1. Synthesis

**Scheme 1:**
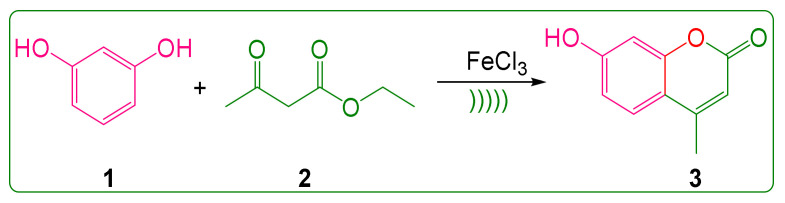
Synthetic protocol of 7-hydroxy-4-methyl-2H-chromen-2-one (**3**).

**Scheme 2:**
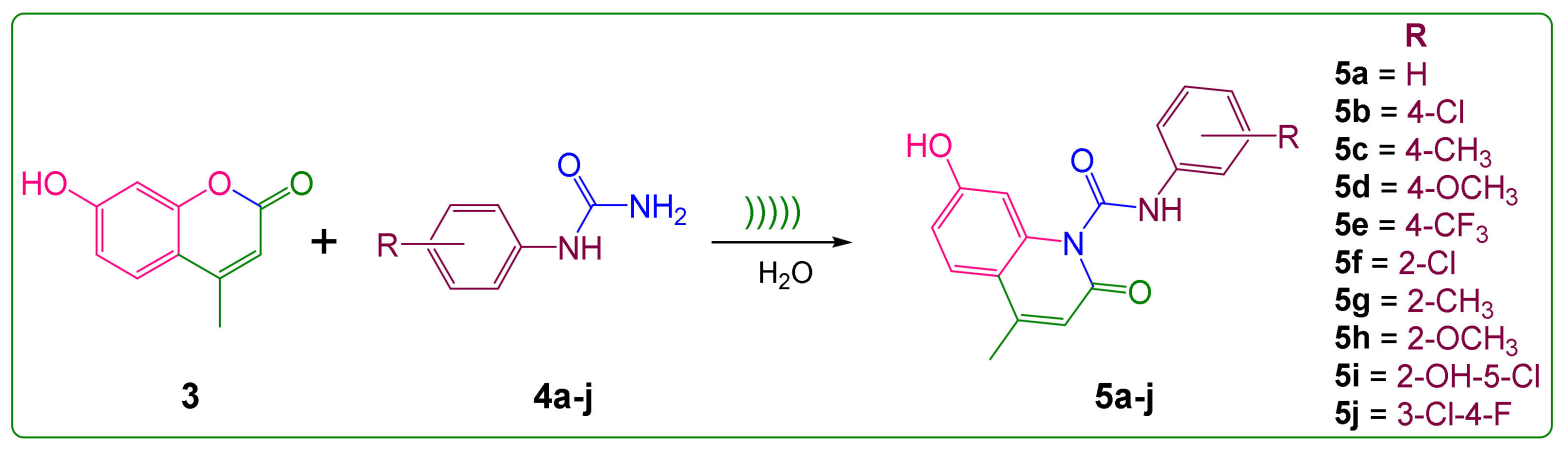
Synthetic protocol of N-aryl-7-hydroxy-4-methyl-2-oxoquinoline-1(2H)-carboxamides (**5a**–**j**).

7-Hydroxy-4-methyl-2H-chromen-2-one (**3**) was initially prepared by ultrasonication from an equimolar mixture of resorcinol (0.05 mol; 5.505 g) and ethyl acetoacetate (0.05 mol; 6.505 g ~6.5 mL) in 10 mL tube adding a catalytic amount of anhydrous FeCl_3_ (0.0025 mol; 41 mg) for 20 min while immersed in cooling bath to obtain a precipitate as per the reported method [37,38]. The synthetic protocol is summarized in Scheme 1. In the subsequent step, an equimolar mixture of 7-hydroxy-4-methyl-2H-chromen-2-one (**3**) (1 mmol; 176 mg) and substituted phenyl urea (**4a**–**j**) (1 mmol) in 10 mL water was ultrasonicated for 20–25 min (20 KHz; 130 W) and the final precipitate was separated by vacuum filtration followed by washing with water and re-crystallized from absolute ethanol to obtain the target title compounds (**5a**–**j**). The substituted phenyl urea (**4a**–**j**) was prepared as per the reported synthetic protocol [39]. The synthetic protocol is summarized in Scheme 2. The synthetic strategy investigated here has the advantages of being easy to implement and having a high conversion rate with swift reaction times in green solvent under ultrasound irradiation, as seen in our previous work [40]. Infrared (IR), nuclear magnetic resonance (^1^H and ^13^C-NMR), and mass spectral data were used to confirm the structure of the final compounds (**5a**–**j**).

### 2.2. Optimization of Reaction Conditions

The reaction conditions were optimized in various solvents, prior to synthesis of compound **5a**, in order to choose a suitable solvent. An equimolar amount of compound **3** (1 mmol; 176 mg) and phenyl urea **4a** (1 mmol; 136 mg) was subjected to ultrasound irradiation in different solvents and the yields were analyzed. The results of optimization of reaction condition are given in Table 1. The yields of the compound were found to be less in ethylacetate (34%; entry 1), acetonitrile (34%; entry 6), dioxane (36%; entry 5), toluene (44%; entry 4), and dimethyl sulfoxide (49%; entry 8). The yields were increased in the solvents like dichloromethane (59%; entry 1), ethanol (64%; entry 2), glacial acetic acid (65%; entry 9) and methanol (69%; entry 3). Except for yield variations, ultrasound-mediated synthesis was found to be uniform in all solvents. The solvent-free synthesis was found to be interesting (71%; entry 10) but it required an elevated temperature (200 °C) and 60 min to complete the reaction. The reaction time was increased to 4 h when the reaction mixture was refluxed in water, but the yield was found to be advantageous (82%; entry 11). Furthermore, when the same reaction was carried out in water under ultrasonication, the yield (91%; entry12) was increased, while the reaction time was lowered to 20 min.

### 2.3. Exploration of Methodology

As a result, we investigated and compared the scope of this synthetic method under optimize conditions in both at the elevated temperature (200 °C) in solvent free conditions, as well as under ultrasonicated modes in water as a green solvent. The physical constants and comparative yields are given in Table 2. The ultrasound-mediated reaction was found to be more beneficial than conventional heating.

### 2.4. Antiproliferative Activity

**Figure 2:**
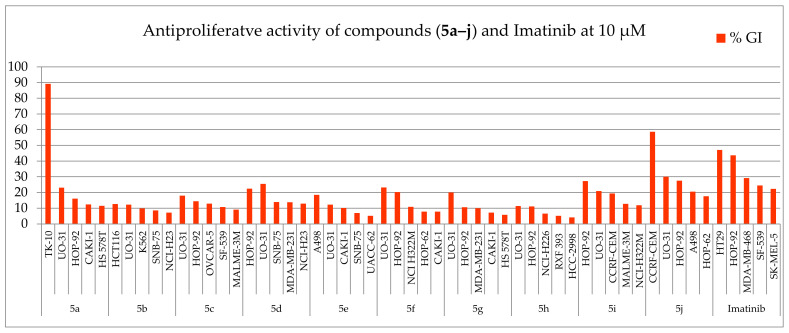
Antiproliferative activity of compounds **5a**–**j** and Imatinib at 10 µM in terms of %GIs.

All the synthetic compounds (**5a**–**l**) were evaluated for their antiproliferative activity in one dose assay against 60 NCI cancer cell line at 10 µM as per the NCI protocols [42,43,44,45]. The results of antiproliferative activity are given in Table 3. The compounds **5c**, **5f**, **5g**, and **5h** showed maximum sensitivity against UO-31 (renal cancer) with percent growth inhibitions (%GIs) of 17.96, 23.13, 19.94, and 11.38, respectively. The compounds **5b**, **5d**, **5e**, and **5i** displayed sensitivity against various cancer cell lines with %GIs of 12.64 (HCT116; colon cancer), 22.47 (HOP-92; non-small cell lung cancer), 18.48 (A498; renal cancer), and 27.21 (HOP-92; non small cell lung cancer), respectively. The compound **5a** displayed maximum sensitivity against TK-10 (renal cancer), UO-31 (renal cancer), HOP-92 (non-small cell lung cancer), CAKI 1 (renal cancer) and HS 578T (breast cancer) with %GIs of 82.90, 23.10, 16.00, 12.39, and 11.50, respectively. Similarly, the compound **5j** displayed CCRF-CEM (leukemia), UO-31 (renal cancer), HOP-92 (non-small cell lung cancer), A498 (renal cancer) and HOP-62 (non-small cell lung cancer) with %GIs of 58.61, 30.00, 27.44, 20.53, and 17.56, respectively. The maximum sensitivity was observed against TK-10 (with compound **5a**) and CCRF-CEM (with compound **5j**). The antiproliferative activity is represented in Figure 2.

### 2.5. Antioxidant Activity

The 1,1-diphenyl-2-picrylhydrazyl (DPPH) free radicals (FR) scavenging activity was performed for some of the target compounds to evaluated antioxidant activity, as per reported protocol [46]. The compound **5a** showed the most promising antioxidant activity with an IC_50_ value of 14.16 ± 0.42 µM comparable to that of the standard ascorbic acid followed by the compound **5d** with an IC_50_ value of 22.90 ± 0.89 µM. The rest of the three compounds (**5b**, **5f**, **5g**, **5h** and **5j**) showed moderate antioxidant activity with IC_50_ values ranging between 24.18 ± 0.41 and 54.45 ± 0.95 µM. The antioxidant activity of five compounds is shown in Table 4.

### 2.6. Molecular Docking

**Figure 3:**
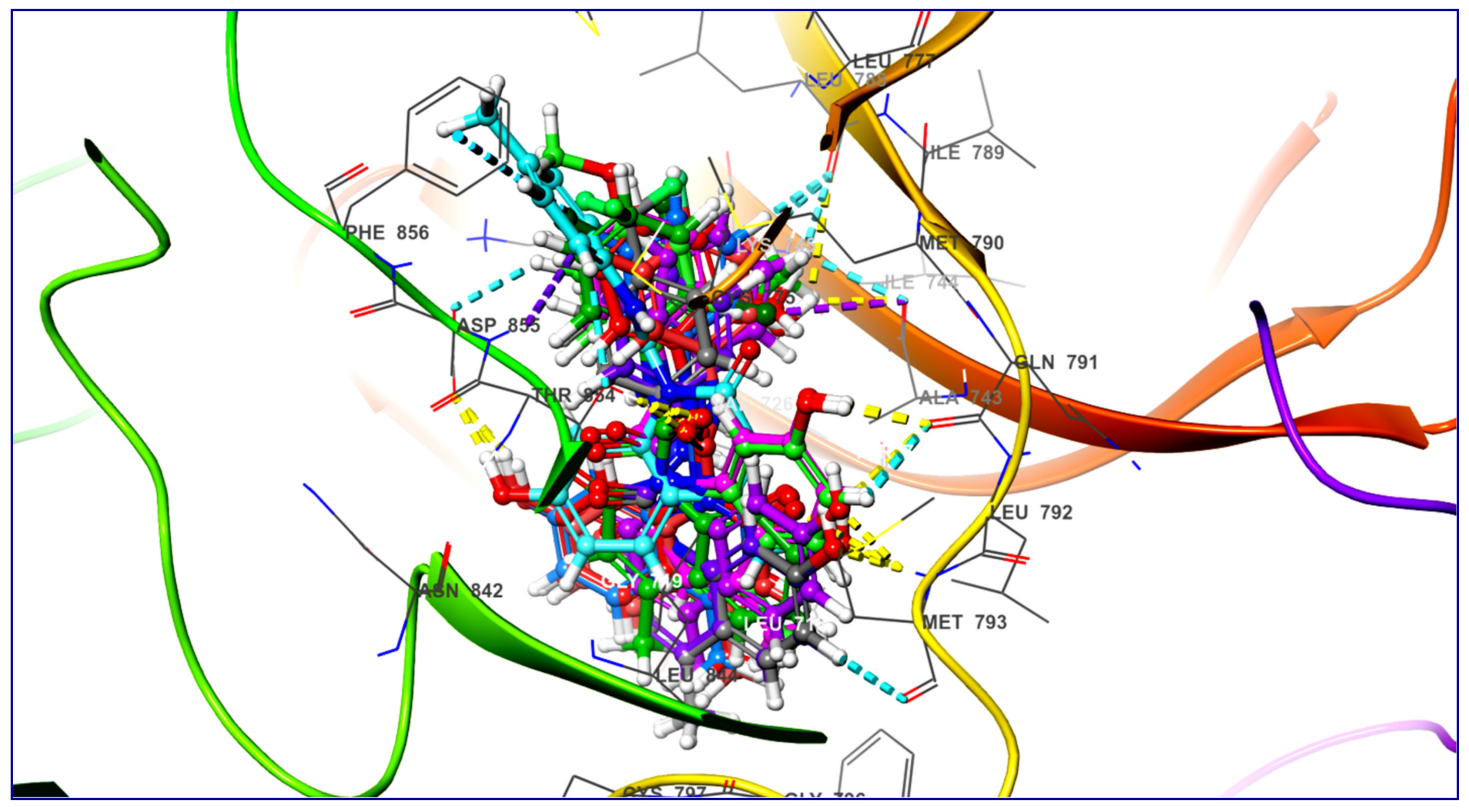
The molecular docking of ligands (**5a**–**j**) against EGFR (PDB ID: 3W2R) showing various types of interactions with the amino acid residues.

**Figure 4:**
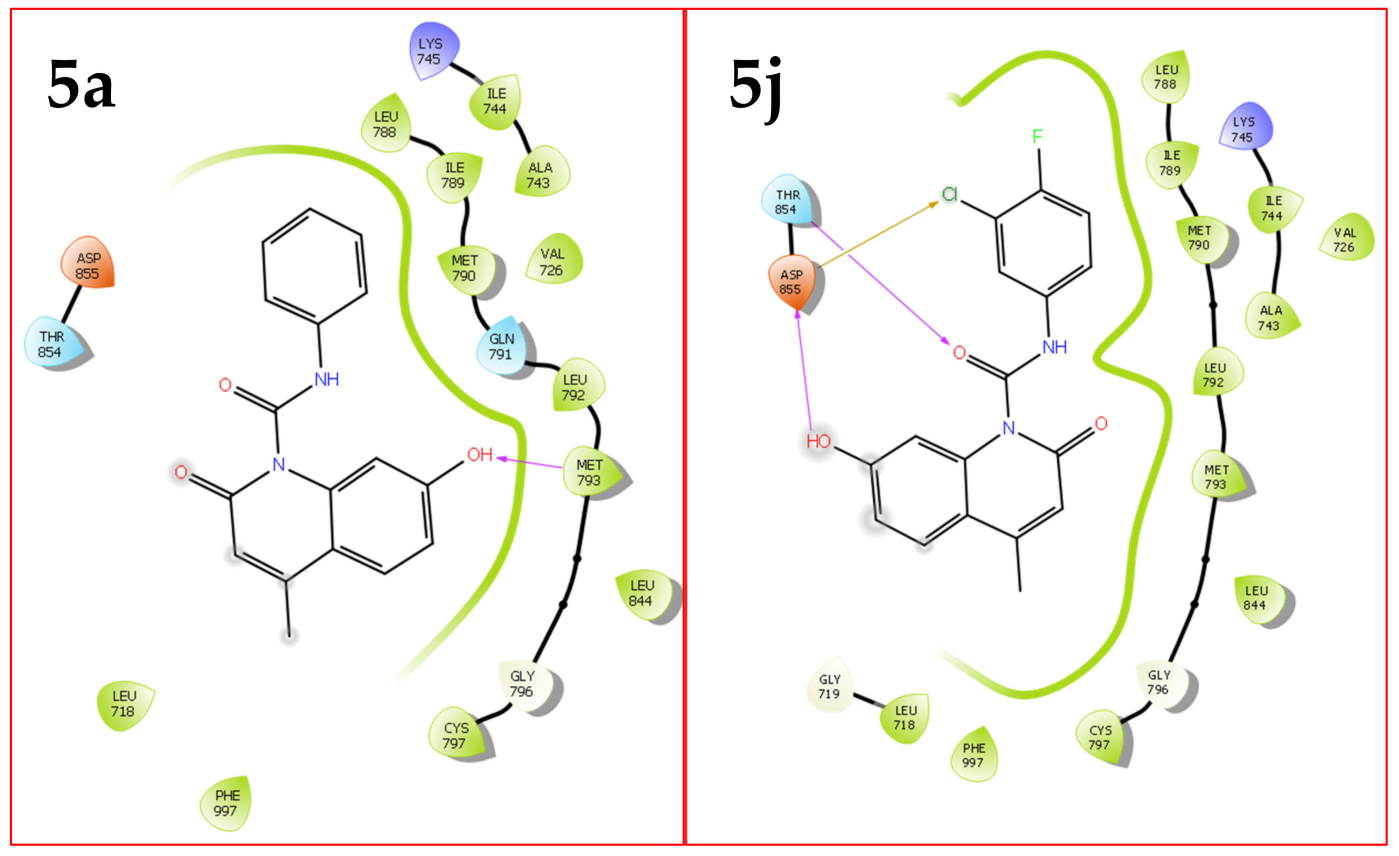
The 2D interactions of ligands **5a** and **5j** within the active site of EGFR (PDB ID: 3W2R).

**Figure 5:**
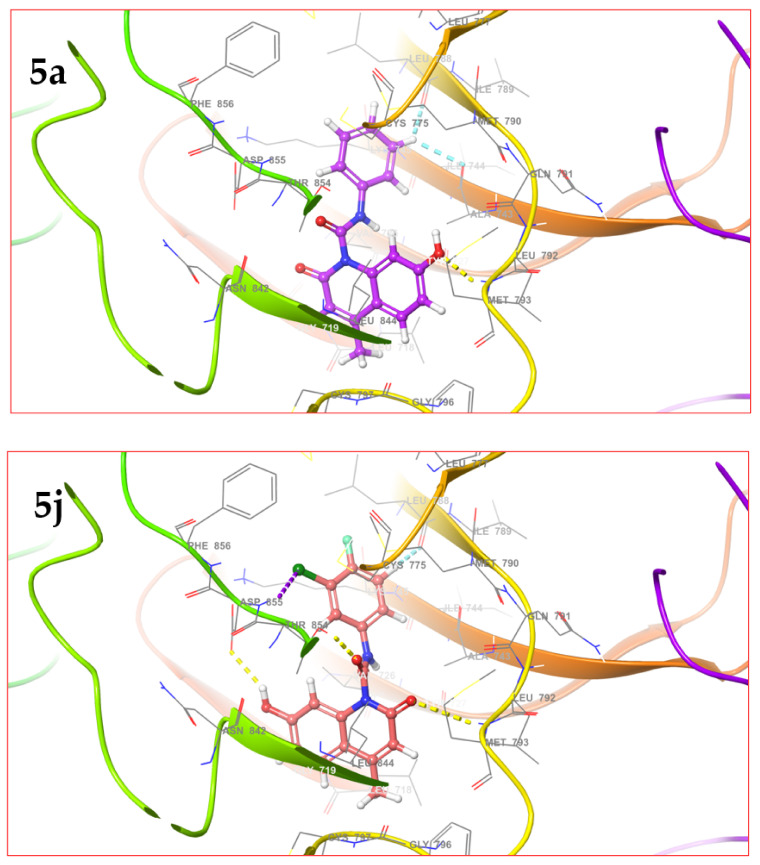
The 3D interactions of ligands **5a** and **5j** within the active site of EGFR (PDB ID: 3W2R).

The molecular docking studies of ligands **5a**–**j** were carried out against EGFR (PDB ID: 3W2R) to identify the putative mechanism of action of these ligands against cancer [47,48]. The ligands showed different types of interaction the amino acid residues within the active site of EGFR include H-bond, π-π-stacking, π-cationic and halogen bond interaction, as shown in Figure 3. The various types of interaction with the amino acid residues are shown in Table 5. The ligands **5a** and **5j** were found to have significant antiproliferative activity. The ligand **5a** showed a H-bond with the residue Met793 with the phenolic function of coumarin ring, while π-π-stacking was observed between the N-phenyl ring and the residues Leu788 and Ala743 (Figure 4 and Figure 5). The ligand **5j** showed two H-bonds, one between the phenolic function of coumarin and the residue Asp855 and another between the carbonyl function (of amide) and the residue Thr854. The ligand **5j** also displayed a π-π-stacking (of phenyl ring) with the residue Leu788 and a halogen bond interaction of 3-chloro function with the residue Asp855 (Figure 4 and Figure 5). The docking images of the rest of the compounds are given in Figures S1 and S2 (Supplementary Materials).

**Figure 6:**
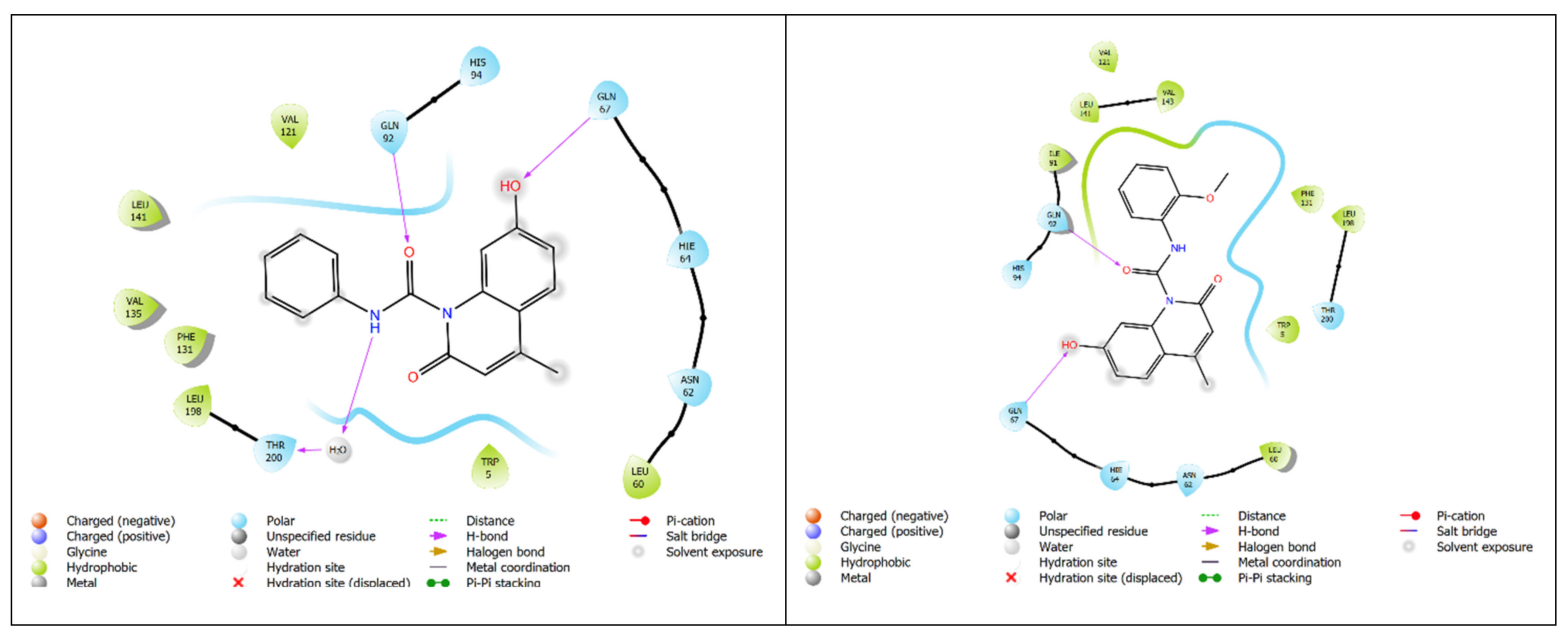
The 2D interactions of ligands **5a** and **5h** within the active site of CA IX (PDB ID: 3DC3).

Similarly, CA was found to be linked with the proliferation of some of the cancer cells due to hypoxia [35,36]. Since the compounds showed anticancer as well as antioxidant activities, docking against CA isozyme IX (PDB ID: 3DC3) was also performed and the docking scores are given in Table S1 (Supplementary Materials) [49]. The ligands showed different types of interaction with the amino acid residues within the active site of CA include H-bond, π-π-stacking, π-cationic and halogen bond interaction. The ligand **5a** showed efficient binding (docking score = −5.337) and displayed three H-bond interations with residues Gln67 (with phenolic function of coumarin), Gln92 (with carbonyl function) and Thr200 (with NH function, through the water molecule). Similarly, the ligand **5h** (docking score = −5.643) displayed two H-bond interations with residues Gln67 (with phenolic function of coumarin), and Gln92 (with carbonyl function). The 2D binding interactions of ligands **5a** and **5h** are shown in Figure 6, while the 2D binding interaction of ligand **5g** is shown in Figure S3 (Supplementary Materials).

### 2.7. ADME Studies

**Figure 7:**
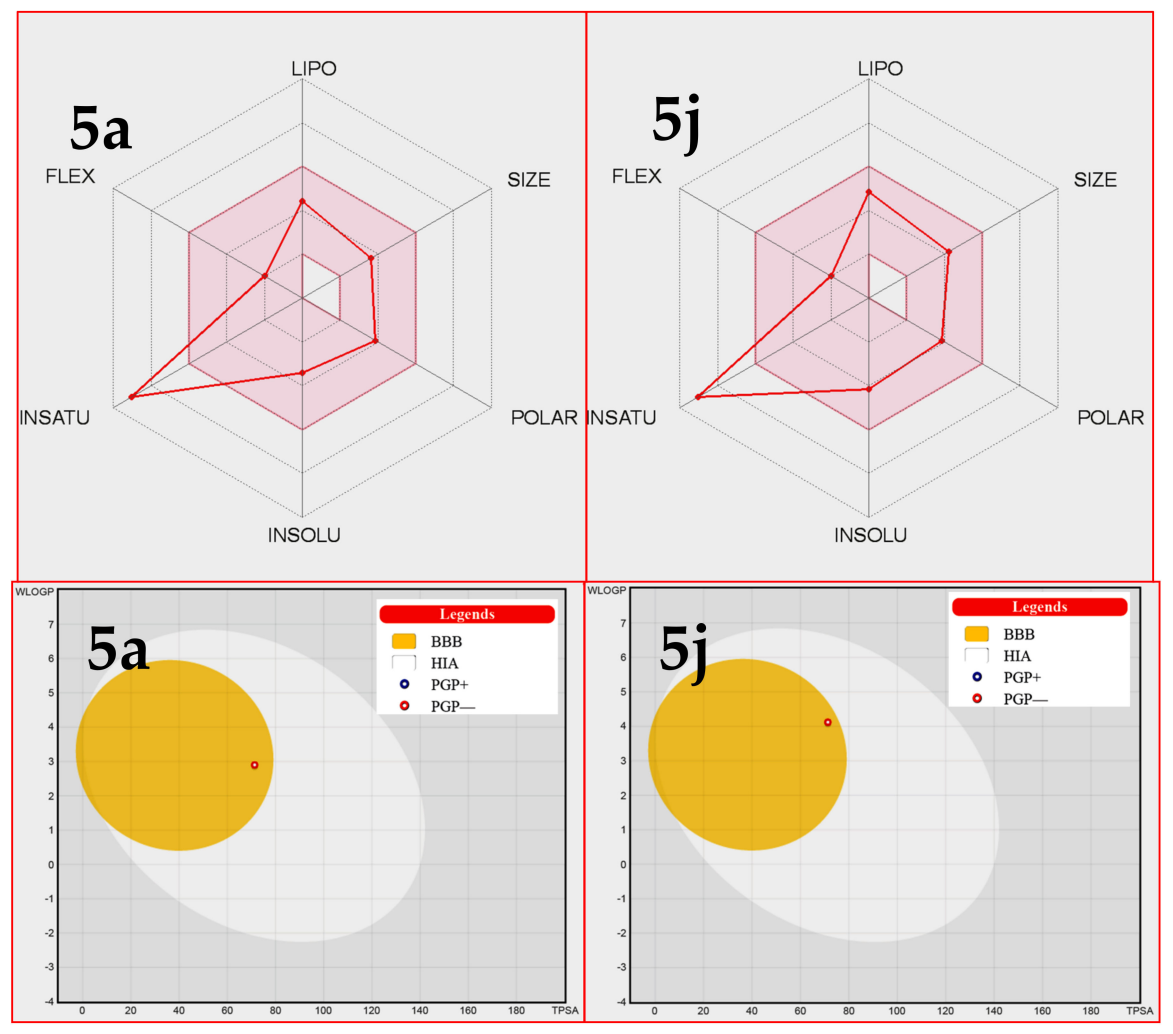
The bioavailability radar plots and boiled-egg graphs for the compounds **5a** and **5j** using Swiss ADME software.

ADME studies are necessary for a drug’s successful absorption from the gastrointestinal system, as well as its oral bioavailability [50,51,52,53,54]. The physicochemical and pharmacokinetic parameters were studied by freely available ADME software [50]. All the compound **5a**–**j** displayed a permissible number of H-bond acceptors (<10), H-bond donors (<5), rotatable bonds (<10) and log P (<5), as shown in Table 6. None of the compounds violated the Lipinski rule of 5, making them promising agents [55]. The most promising compounds (**5a** and **5j**) displayed promising physicochemical and pharmacokinetic parameters in ADME prediction studies. These compounds (**5a** and **5j**) displayed high gastrointestinal (GI) absorption (as seen in the radar plots) as well as their ability to cross the blood–brain barrier (BBB) (as seen in boiled egg diagram) (as shown in Figure 7) together with zero pan assay interference compounds (PAINS) prediction [56].

## 3. Discussion

Quinolines are a class of bicyclic hetero-aromatic compound with a wide range of biological activities. Many of the anticancer quinolines are in clinical trials or are in the early stages of development [32]. In the current study, a green approach was used for the synthesis of oxoquinolines (**5a**–**j**). All of the quinoline analogues followed the Lipinski rule of five and ADME predictions, indicating that they could be used as oral drugs. There were three to four numbers of rotatable bonds, making the compound flexible enough to bind with molecular target. Compounds **5a** and **5j** were discovered to be capable of crossing the blood–brain barrier, as shown in the boiled egg representations and bioavailability radar plots (Figure 7). However, none of the compounds were recognized as PAINS materials in SWISS ADME prediction studies. All these compounds were synthesized using a green approach. We attempted the synthesis of oxoquinolines in several solvents, with the best results obtained in water. Water has various advantages because it is a low-cost, readily available, non-toxic, and non-flammable solvent that is both economically and environmentally acceptable [28]. Using water as a solvent, several research groups successfully synthesized quinoline, a bicyclic heterocycle [23,29,30,31]. Similarly, when compared to conventional synthesis, ultrasound-mediated synthesis is consistently found to be superior in terms of yields and reaction times [23,37].

The antiproliferative activity of the compounds (**5a**–**j**) was carried out at 10 µM on nearly five dozen cancer cell lines and the results were represented as GP as well as %GIs. The GP and %GI are correlated as %GI = 100 − GP. In this study the compounds **5a** and **5j** showed promising anticancer activity with maximum sensitivity against TK-10 (%GI = 82.90) and CCRF-CEM (%GI = 58.61) cancer cell lines respectively. The 3-chloro-4-flouro substitution on *N*-phenyl ring of compound **5j** demonstrated maximum antiproliferative activity. Similarly, the same function displayed halogen bond (of 3-chloro function) with the residue Asp855, apart from H-bond (Asp855 and Thr854) as well as π-π-stacking (Leu788) present in the active site of EGFR. The compound **5a** displayed the most promising antiproliferative activity against TK-10 (renal cancer), displayed two types of interactions [H-bond (Met793), π-π-stacking (Leu788 and Ala743)] in molecular docking studies. The compound **5g** (UO-31; %GI = 19.94), **5h** (UO-31; %GI = 11.38) and **5i** (HOP-92; %GI = 27.21) displayed only H-bond interaction in the molecular docking studies. The average GP of the compounds was compared in order to assess the structure-activity relationship (SAR). The chloro function at meta (**5j**) and ortho (**5f**) position displayed halogen bond interaction, while para (**5b**) substitution didn’t displayed halogen bond interaction. The order of antiproliferative activity followed with substitutions as 3-Cl-4-F ≥ H > 2-OH-5-Cl > 2-Cl > 4-OCH_3_ > 4-CH_3_ > 4-Cl > 4-CF_3_ > 2-CH_3_.

Compound **5a** also demonstrated promising DPPH free radical scavenging activity with an IC_50_ value of 14.16 ± 0.42 µM, while compound **5d** showed antioxidant activity with and IC_50_ value of 22.90 ± 0.89 µM and rest of the compounds showed moderate antioxidant activity. Antioxidant supplementation appears to be an essential step in preventing or reducing oxidative damage associated with high altitude exposure [57,58]. The literature survey revealed that acetazolamide (a specific inhibitor of carbonic anhydrase), is used to treat high altitude pulmonary oedema (HAPE) [59,60]. The ligands **5a**, **5g**, and **5h** efficiently bind within the active site of CA.

## 4. Materials and Methods

### 4.1. Synthesis

An equimolar mixture of 7-hydroxy-4-methyl-2*H*-chromen-2-one (**3**) (1 mmol; 176 mg) and substituted phenyl urea (**4a**–**j**) (1 mmol) in 10 mL water was ultrasonicated at 130 W for 20–45 min. After the total consumption of the reactants the resultant precipitate was filtered under vacuum washed with water and collected. The crude product was further re-crystallized with absolute ethanol to obtain *N*-substituted phenyl-7-hydroxy-4-methyl-2-oxoquinoline-1(2*H*)carboxamides (**5a**–**j**) [40]. 7-Hydroxy-4-methyl-2*H*-chromen-2-one (**3**) and substituted phenyl urea (**4a**–**j**) was synthesized as per the reported methods [37,39]. The characterization data of synthesized compounds are given in the Supplementary Materials.

### 4.2. Antiproliferative Activity

The target compounds (**5a**–**m**) were tested at 10 µM for their antiproliferative activity against nine different panels of 60 cancer cell lines. It was measured at the National Cancer Institute using the Sulforhodamine B (SRB) assay (NCI US) [42,43,44,45].

### 4.3. Antioxidant Activity

The 1,1-diphenyl-2-picrylhydrazyl (DPPH) free radicals (FR) scavenging activity was carried out according to the standard protocol reported to evaluate the antioxidant activity of the some of the target compounds [46].

### 4.4. Molecular Docking Studies

The molecular docking against EGFR was performed for the ligands, **5a**–**j**. The EGFR (PDB: 3W2R) X-ray crystal structure with a resolution of 2.05 Å; R-value 0.220 (observed) was obtained from the protein data bank [47]. The ligands **5a**–**j** were saved as a mol file and the docking was done as per the reported protocol [48].

Similarly, the molecular docking against CA was performed for the ligands, **5a**–**j**. The CA (PDB: 3DC3) X-ray crystal structure with a resolution of 1.70 Å; R-value 0.141 (work) was obtained from the protein data bank [49]. The ligands **5a**–**j** were saved as a mol file and the docking was done as per the reported protocol [35].

### 4.5. ADME Studies

The pharmacokinetic and physicochemical parameters were studied by freely available ADME prediction software [50]. The various parameters include number of H-bond donors, number of H-bond acceptors, number of rotatable bonds, lipophilicity, bioavailability, GI absorption, BBB permeation, PAINS alert and P-pg substrate.

## 5. Conclusions

Following ultrasound irradiation, we successfully prepared oxoquiolines (**5a**–**j**) in high yield in green solvent water. All of these compounds adhered to the Lipinski rule of five, as well as physicochemical and pharmacokinetic parameters as measured by ADME software. The antiproliferative activity was carried out against 60 NCI cancer cell lines at 10 µM. Compounds **5a** and **5j** demonstrated the most promising activity against TK-10 (82.90% growth inhibition) and CCRF-CEM (58.61% growth inhibition) cell lines respectively. Compound **5d** also demonstrated promising DPPH free radical scavenging activity with an IC_50_ value of 14.16 ± 0.42 µM. Three types of interactions were observed in molecular docking studies: H-bond, π-π-stacking, and halogen bond interactions. The reported work could be expanded through molecular modification, which would be extremely beneficial to researchers working on anticancer research programs. Since the compounds showed significant anticancer and antioxidant activities, the title compounds were further studied for their CA inhibitory activity. Some of the ligands (**5a**, **5g**, and **5h**) displayed efficient binding with CA in the in-silico studies. The antioxidant activity of compounds **5a** and **5h** were found to be promising.

Some of the compounds displayed promising anticancer and antioxidant activities. Furthermore, all the ligands showed efficient binding within the active sites of EGFR, while some of the ligands efficiently bind within the active site of the CA in-silico studies. Antioxidant supplementation appears to be an essential step in preventing or reducing oxidative damage associated with high altitude exposure. As a result, the current discovery could aid in the development of novel therapeutic compounds for the treatment of cancer, oxidative stress, and other high-altitude disorders.

## Figures and Tables

**Table 1:** Optimization of reaction conditions for the synthesis of N-phenyl-7-hydroxy-4-methyl-2-oxoquinoline-1(2H)carboxamide (**5a**).

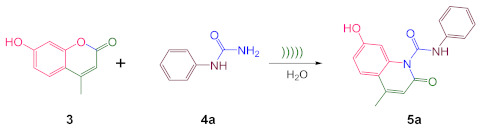				
Entry	Reflux/)))))/Fused	Solvent	Reaction Time	Yield ^b^ (%)
1	)))))	Ethylacetate	20 min	34
2	)))))	Methanol	20 min	60
3	)))))	Ethanol	20 min	67
4	)))))	Toluene	20 min	44
5	)))))	Dioxane	20 min	36
6	)))))	Acetonitrile	20 min	54
7	)))))	Dichloromethane	20 min	59
8	)))))	Dimethyl sulfoxide	20 min	49
9	)))))	Glacial acetic acid	20 min	65
10	Fused at 200 °C	Solvent free	60 min	71
11	Reflux	Water	240 min	82
12	)))))	Water	20 min	91

Reaction condition: 7-hydroxy-4-methyl-2H-chromen-2-one (**3**) (1 mmol; 176 mg) and phenyl urea (**4a**) (1 mmol; 136 mg); ^b^ Yield of final dried compounds.

**Table 2:** Physical constants and yields of the prepared oxoquinolines (**5a**–**j**).

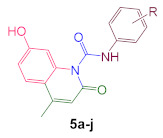						
S. No.	Compound	R	Mp (ºC)	R_f_ *	Yield ^a^ (Time in min)	
					Fused at 200 °C ^b^	)))))) ^c^
1	**5a**	H	78–80	0.66	82% (60 min)	91% (20 min)
2	**5b**	4-Cl	160–162	0.62	72% (60 min)	90% (20 min)
3	**5c**	4-CH_3_	140–142	0.71	60% (60 min)	81% (5 min)
4	**5d**	4-OCH_3_	128–130	0.69	58% (60 min)	80% (20 min)
5	**5e**	4-CF_3_	142–144	0.63	71% (60 min)	90% (20 min)
6	**5f**	2-Cl	152–154	0.68	62% (60 min)	91% (20 min)
7	**5g**	2-CH_3_	102–104	0.72	68% (60 min)	78% (20 min)
8	**5h**	2-OCH_3_	110–112	0.78	69% (60 min)	80% (20 min)
9	**5i**	2-OH-5-Cl	156–158	0.74	56% (60 min)	70% (25 min)
10	**5j**	3-Cl-4-F	180–182	0.64	84% (60 min)	92% (20 min)

* Benzen: acetone (9:1); ^a^ Yield of final dried compounds; ^b^ Reaction condition: 7-hydroxy-4-methyl-2*H*-chromen-2-one (**3**) (1 mmol; 176 mg) and substituted phenyl urea (**4a**–**j**) (1 mmol); 200 °C (fusion) [41]; ^c^ Reaction condition: 7-hydroxy-4-methyl-2*H*-chromen-2-one (**3**) (1 mmol; 176 mg) and substituted phenyl urea (**4a**–**j**) (1 mmol); H_2_O 10 mL; ))))) (Ultrasound) 20 KHz; 130 W.

**Table 3:** The antiproliferative activity of compounds **5a**–**j** at 10 µM.

Compound/NSC Code	Cancer Cell Lines Assay in Single Dose Assay 10 µM Concentration		
	The Most Sensitive Cell Lines	GP	%GI
**5a** **NSC** 805519	TK-10 (Renal Cancer)	17.10	82.90
	UO-31 (Renal Cancer)	76.90	23.10
	HOP-92 (Non Small Cell Lung Cancer)	84.00	16.00
	CAKI 1 (Renal Cancer)	87.61	12.39
	HS 578T (Breast Cancer)	88.50	11.50
**5b** **NSC** 805508	HCT116 (Colon Cancer)	87.36	12.64
	UO-31 (Renal Cancer)	87.82	12.18
	K562 (Leukemia)	90.23	9.77
	SNB-75 (CNS Cancer)	91.49	8.51
	NCI-H23 (Non-Small Cell Lung Cancer)	92.81	7.19
**5c** **NSC** 805512	UO-31 (Renal Cancer)	82.04	17.96
	HOP-92 (Non Small Cell Lung Cancer)	85.60	14.40
	OVCAR-5 (Ovarian Cancer)	87.13	12.87
	SF-539 (CNS Cancer)	89.28	10.72
	MALME-3M (Melanoma)	90.95	9.05
**5d** **NSC** 805516	HOP-92 (Non Small Cell Lung Cancer)	77.53	22.47
	UO-31 (Renal Cancer)	74.56	25.44
	SNB-75 (CNS Cancer)	86.10	13.90
	MDA-MB-231/ATCC (Breast Cancer)	86.25	13.75
	NCI-H23 (Non-Small Cell Lung Cancer)	87.17	12.83
**5e** **NSC** 805511	A498 (Renal Cancer)	81.52	18.48
	UO-31 (Renal Cancer)	87.76	12.24
	CAKI-1 (Renal Cancer)	89.97	10.03
	SNB-75 (CNS Cancer)	93.09	6.91
	UACC-62 (Melanoma)	94.90	5.10
**5f** **NSC** 805517	UO-31 (Renal Cancer)	76.87	23.13
	HOP-92 (Non-Small Cell Lung Cancer)	79.74	20.26
	NCI-H322M (Non Small Cell Lung Cancer)	88.75	10.79
	HOP-62 (Non-Small Cell Lung Cancer)	89.21	7.79
	CAKI-1 (Renal Cancer)	92.27	7.73
**5g** **NSC** 805513	UO-31 (Renal Cancer)	80.06	19.94
	HOP-92 (Non-Small Cell Lung Cancer)	89.43	10.57
	MDA-MB-231/ATCC (Breast Cancer)	90.02	9.98
	CAKI-1 (Renal Cancer)	92.87	7.13
	HS 578T (Breast Cancer)	94.23	5.77
**5h** **NSC** 805515	UO-31 (Renal Cancer)	88.62	11.38
	HOP-92 (Non-Small Cell Lung Cancer)	88.90	11.10
	NCI-H226 (Non-Small Cell Lung Cancer)	93.46	6.54
	RXF 393 (Renal Cancer)	94.83	5.17
	HCC-2998 (Colon Cancer)	95.92	4.08
**5i** **NSC** 805520	HOP-92 (Non-Small Cell Lung Cancer)	72.79	27.21
	UO-31 (Renal Cancer)	79.09	20.91
	CCRF-CEM (Leukemia)	80.64	19.36
	MALME-3M (Melanoma)	87.21	12.79
	NCI-H322M (Non-Small Cell Lung Cancer)	88.16	11.84
**5j** **NSC** 805518	CCRF-CEM (Leukemia)	41.39	58.61
	UO-31 (Renal Cancer)	70.00	30.00
	HOP-92 (Non Small Cell Lung Cancer)	72.56	27.44
	A498 (Renal Cancer)	79.47	20.53
	HOP-62 (Non Small Cell Lung Cancer)	82.44	17.56
Imatinib * NSC 759854	HT29 (Colon Cancer)	52.9	47.1
	HOP-92 (Non-Small Cell Lung Cancer)	56.3	43.7
	MDA-MB-468 (Breast Cancer)	70.9	29.1
	SF-539 (CNS Cancer)	75.5	24.5
	SK-MEL-5 (Melanoma)	77.7	22.3

* The anticancer data of Imatinib was retrieved from National Cancer Institute database with NSC code 759854 [42].

**Table 4:** The antioxidant activity of some of target compounds.

S. No.	Compound	Free Radical Scavenging Activity IC_50_ (μM)
1	**5a**	14.16 ± 0.42
2	**5b**	24.18 ± 0.41
3	**5d**	22.90 ± 0.89
4	**5f**	36.32 ± 0.93
5	**5g**	54.45 ± 0.95
6	**5h**	24.52 ± 0.72
7	**5j**	31.11 ± 0.91
8	Ascorbic acid	13.99 ± 0.89

**Table 5:** The molecular docking results of compounds **5a**–**j** against EGFR.

S. No.	Ligand	Docking Score	Types of Interaction
1	**5a**	−8.839	H-bond (Met793), π-π-Stacking (Leu788 and Ala743)
2	**5b**	−9.013	H-bond (Met793), π-π-Stacking (Met793)
3	**5c**	−7.944	H-bond (Asp855), π-π-Stacking (Thr854 and Phe856)
4	**5d**	−8.216	H-bond (Gln791), π-π-Stacking (Gln791 and Asp855)
5	**5e**	−9.378	H-bond (Met793), π-π-Stacking (Met793)
6	**5f**	−8.797	H-bond (Gln791), Halogen bond (Ala743), π-π-Stacking (Gln791)
7	**5g**	−8.714	H-bond (Asp855 and Thr854)
8	**5h**	−9.416	H-bond (Met793, Gln791 and Thr854)
9	**5i**	−9.013	H-bond (Asp855, Ala743 and Thr854)
10	**5j**	−8.305	H-bond (Asp855 and Thr854), Halogen bond (Asp855), π-π-Stacking (Leu788)

**Table 6:** The ADME studies of compounds **5a**–**j**.

Parameters	Compounds									
	5a	5b	5c	5d	5e	5f	5g	5h	5i	5j
No. H-bond acceptor	3	3	3	4	6	3	3	4	4	4
No. H-bond donor	2	2	2	2	2	2	2	2	3	2
LogP_O/W_(iLOGP)	1.82	2.39	2.02	2.40	2.58	2.22	2.13	2.15	2.00	2.62
No. rotatable bonds	3	3	3	4	4	3	3	4	3	3
TPSA	71.33	71.33	71.33	80.56	71.33	71.33	71.33	80.56	91.56	71.33
Log K_P_ (skin permeation)	−6.50	−6.27	−6.33	−6.70	−6.29	−6.27	−6.33	−6.70	−6.61	−6.31
Lipinski’s rule violation	No	No	No	No	No	No	No	No	No	No
Bioavailability score	0.55	0.55	0.55	0.55	0.55	0.55	0.55	0.55	0.55	0.55
GI absorption	High	High	High	High	High	High	High	High	High	High
BBB permeation	Yes	Yes	Yes	No	No	Yes	Yes	No	No	Yes
PAINS alerts	0	0	0	0	0	0	0	0	0	0
P-pg substrate	No	No	No	No	No	No	No	No	No	No

## Data Availability

The authors confirm that the data supporting the study’s findings are included in the article and its Supplementary Materials.

## Supplementary Materials

Supplementary materials containing characterization of compounds (**5a**–**j**) are available online; Figure S1: The 2D interactions of ligands within the active site of EGFR; Figure S2: The 3D interactions of ligands within the active site of EGFR; Figure S3: The 3D interactions of ligand **5g** within the active site of CA IX; Table S1: The molecular docking results of compounds **5a**–**j** against CA IX (PDB ID: 3DC3).

## Sample Availability

Samples of the compounds not available.

## References

[1] Nita M., Grzybowski A. (2016). The Role of the Reactive Oxygen Species and Oxidative Stress in the Pathomechanism of the Age-Related Ocular Diseases and Other Pathologies of the Anterior and Posterior Eye Segments in Adults. Oxid. Med. Cell. Longev..

[2] Dosek A., Ohno H., Acs Z., Taylor A.W., Radak Z. (2007). High altitude and oxidative stress. Respir. Physiol. Neurobiol..

[3] Zuo L., Hallman A.H., Yousif M.K., Chien M.T. (2012). Oxidative stress, respiratory muscle dysfunction, and potential therapeutics in chronic obstructive pulmonary disease. Front. Biol..

[4] Dhalla N.S., Temsah R.M., Netticadan T. (2000). Role of oxidative stress in cardiovascular diseases. J. Hypertens..

[5] Roberts C.K., Sindhu K.K. (2009). Oxidative stress and metabolic syndrome. Life Sci..

[6] Sorce S., Krause K.-H. (2009). NOX Enzymes in the Central Nervous System: From Signaling to Disease. Antioxid. Redox Signal..

[7] Liao D., Corle C., Seagroves T., Johnson R.S. (2007). Hypoxia-Inducible Factor-1α Is a Key Regulator of Metastasis in a Transgenic Model of Cancer Initiation and Progression. Cancer Res..

[8] Uttara B., Singh A.V., Zamboni P., Mahajan R.T. (2009). Oxidative Stress and Neurodegenerative Diseases: A Review of Upstream and Downstream Antioxidant Therapeutic Options. Curr. Neuropharmacol..

[9] Frijhoff J., Winyard P.G., Zarkovic N., Davies S.S., Stocker R., Cheng D., Knight A.R., Taylor E.L., Oettrich J., Ruskovska T. (2015). Clinical Relevance of Biomarkers of Oxidative Stress. Antioxid. Redox Signal..

[10] Chen K., Lu P., Beeraka N.M., Sukocheva O.A., Madhunapantula S.V., Liu J., Sinelnikov M.Y., Nikolenko V.N., Bulygin K.V., Mikhaleva L.M. (2020). Mitochondrial mutations and mitoepigenetics: Focus on regulation of oxidative stress-induced responses in breast cancers. Semin. Cancer Biol..

[11] Boland M.L., Chourasia A.H., MacLeod K.F. (2013). Mitochondrial Dysfunction in Cancer. Front. Oncol..

[12] (2020). WHO Report on Cancer: Setting Priorities, Investing Wisely and Providing Care for All.

[13] Reuter S., Gupta S.C., Chaturvedi M.M., Aggarwal B.B. (2010). Oxidative stress, inflammation, and cancer: How are they linked?. Free Radic. Biol. Med..

[14] Kharissova O.V., Kharisov B.I., González C.M.O., Méndez Y.P., López I. (2019). Greener synthesis of chemical compounds and materials. R. Soc. Open Sci..

[15] Menges N., Saleh H.E.D., Koller M. (2018). The Role of Green Solvents and Catalysts at the Future of Drug Design and of Synthesis. Green Chemistry.

[16] Dallinger D., Kappe C.O. (2007). Microwave-Assisted Synthesis in Water as Solvent. Chem. Rev..

[17] Zang H., Zhang Y., Zang Y., Cheng B.-W. (2010). An efficient ultrasound-promoted method for the one-pot synthesis of 7,10,11,12-tetrahydrobenzo[c]acridin-8(9H)-one derivatives. Ultrason. Sonochem..

[18] Jarag K., Pinjari D.V., Pandit A.B., Shankarling G. (2011). Synthesis of chalcone (3-(4-fluorophenyl)-1-(4-methoxyphenyl)prop-2-en-1-one): Advantage of sonochemical method over conventional method. Ultrason. Sonochem..

[19] Bakht M.A., Ansari M.J., Riadi Y., Ajmal N., Ahsan M.J., Yar M.S. (2016). Physicochemical characterization of benzalkonium chloride and urea based deep eutectic solvent (DES): A novel catalyst for the efficient synthesis of isoxazolines under ultrasonic irradiation. J. Mol. Liq..

[20] Mason T.J. (2007). Sonochemistry and the environment—Providing a “green” link between chemistry, physics and engineering. Ultrason. Sonochem..

[21] Yadav J., Reddy B., Reddy K.S. (2003). Ultrasound-accelerated synthesis of chiral allylic alcohols promoted by indium metal. Tetrahedron.

[22] Geesi M.H., Moustapha M.E., Bakht M.A., Riadi Y. (2020). Ultrasound-accelerated green synthesis of new quinolin-2-thione derivatives and antimicrobial evaluation against Escherichia coli and Staphylococcus aureus. Sustain. Chem. Pharm..

[23] Geesi M.H., Ouerghi O., Elsanousi A., Kaiba A., Riadi Y. (2020). Ultrasound-Assisted Preparation of Cu-Doped TiO_2_ Nanoparticles as a Nanocatalyst for Sonochemical Synthesis of Pyridopyrimidines. Polycycl. Aromat. Compd..

[24] Rao M.S., Haritha M., Chandrasekhar N., Rao M.V.B., Pal M. (2019). Ultrasound mediated synthesis of 6-substituted 2,3-dihydro-1H-pyrrolo[3,2,1-ij]quinoline derivatives and their pharmacological evaluation. Arab. J. Chem..

[25] Dofe V.S., Sarkate A.P., Shaikh Z.M., Gill C.H. (2017). Ultrasound-Mediated Synthesis of Novel 1,2,3-Triazole-Based Pyrazole and Pyrimidine Derivatives as Antimicrobial Agents. J. Heterocycl. Chem..

[26] Tabassum S., Govindaraju S., Khan R.R., Pasha M.A. (2016). Ultrasound mediated, green innovation for the synthesis of polysub-stituted 1,4-dihydropyridines. RSC Adv..

[27] Castro-Puyana M., Marina M.L., Plaza M. (2017). Water as green extraction solvent: Principles and reasons for its use. Curr. Opin. Green Sustain. Chem..

[28] Simon M.-O., Li C.-J. (2012). Green chemistry oriented organic synthesis in water. Chem. Soc. Rev..

[29] Lee S.Y., Cheon C.-H. (2018). On-Water Synthesis of 2-Substituted Quinolines from 2-Aminochalcones Using Benzylamine as the Nucleophilic Catalyst. J. Org. Chem..

[30] Gopi P., Sarveswari S. (2017). Effective water mediated green synthesis of polysubstituted quinolines without energy expenditure. Mon. Chem. Chem. Mon..

[31] Borah G., Borah P., Bhuyan A., Banik B.K. (2021). Facile Synthesis of Quinolines in Water. Curr. Org. Chem..

[32] Afzal O., Kumar S., Haider R., Ali R., Kumar R., Jaggi M., Bawa S. (2015). A review on anticancer potential of bioactive heterocycle quinoline. Eur. J. Med. Chem..

[33] Wissner A., Overbeek E., Reich M.F., Floyd M.B., Johnson B.D., Mamuya N., Rosfjord E.C., Discafani C., Davis R., Shi X. (2003). Synthesis and structure-activity relationships of 6,7-disubstituted 4-anilinoquinoline-3-carbonitriles. The design of an orally active, irreversible inhibitor of thetyrosine kinase activity of the epidermal growth factor receptor (EGFR) and the human ep-idermal growth factor receptor-2 (HER-2). J. Med. Chem..

[34] Cherian M.A., Ma C.X. (2017). The role of neratinib in HER2-driven breast cancer. Future Oncol..

[35] Genis C., Sippel K.H., Case N., Cao W., Avvaru B.S., Tartaglia L.J., Govindasamy L., Tu C., Agbandje-McKenna M., Silverman D.N. (2009). Design of a Carbonic Anhydrase IX Active-Site Mimic to Screen Inhibitors for Possible Anticancer Properties. Biochemistry.

[36] Supuran C.T., Briganti F., Tilli S., Chegwidden W.R., Scozzafava A. (2001). Carbonic anhydrase inhibitors: Sulfonamides as anti-tumor agents?. Bioorg. Med. Chem..

[37] Ali A., Ali A., Bakht M.A., Ahsan M.J. (2021). Ultrasound promoted synthesis of N-(substituted phenyl)-2-(7-hydroxy-4-methyl-2H-chromen-2-ylidene)hydrazine-1-carboxamides as cytotoxic and antioxidant agents. J. Mol. Struct..

[38] Prousis K.C., Avlonitis N., Heropoulos G.A., Calogeropoulou T. (2014). FeCl3-catalysed ultrasonic-assisted, solvent-free synthesis of 4-substituted coumarins. A useful complement to the Pechmann reaction. Ultrason. Sonochem..

[39] Ahsan M.J., Yadav R.P., Saini S., Hassan M.Z., Bakht M.A., Jadav S.S., Al-Tamimi A.B.S., Geesi M.H., Ansari Y., Khalilullah H. (2017). Synthesis, Cytotoxic Evaluation, and Molecular Docking Studies of New Oxadiazole Analogues. Lett. Org. Chem..

[40] Bakht M.A., Azam F., Ali A., Thomas R., Pooventhiran T., Ali A., Ahsan M.J. (2022). Synthesis and biological studies of oxoquinolines: Experimental and theoretical investigations. J. Mol. Struct..

[41] Ali A., Ali A., Salahuddin, Bakht M.A., Ahsan M.J. (2021). Synthesis and Biological Evaluations of N-(4-Substituted Phenyl)-7-Hydroxy-4-Methyl-2-Oxoquinoline-1(2H)-Carbothioamides. Polycycl. Aromat. Compd..

[42] National Cancer Institute (NCI) DTP Homepage: Cancer Screen 09/2014: NCI-60 DTP Human Tumor Cell Line Screen. The National Cancer Institute (NCI) Is Gratefully Acknowledged for Its Excellent Screening Service. http://dtp.nci.nih.gov.

[43] Monks A., Scudiero D., Skehan P., Shoemaker R., Paull K., Vistica D., Hose C., Langley J., Cronise P., Vaigro-Wolff A. (1991). Feasibility of a High-Flux Anticancer Drug Screen Using a Diverse Panel of Cultured Human Tumor Cell Lines. JNCI J. Natl. Cancer Inst..

[44] Boyd M.R., Paull K.D. (1995). Some practical considerations and applications of the National Cancer Institute in vitro anticancer drug discovery screen. Drug Dev. Res..

[45] Shoemaker R.H. (2006). The NCI60 human tumour cell line anticancer drug screen. Nat. Rev. Cancer.

[46] Koleva I.I., Van Beek T.A., Linssen J.P.H., De Groot A., Evstatieva L.N. (2002). Screening of Plant Extracts for Antioxidant Activity: A Comparative Study on Three Testing Methods. Phytochem. Anal..

[47] PDB of EGFR. https://www.rcsb.org/structure/3W2R.

[48] Sogabe S., Kawakita Y., Igaki S., Iwata H., Miki H., Cary D.R., Takagi T., Takagi S., Ohta Y., Ishikawa T. (2012). Structure-Based Approach for the Discovery of Pyrrolo[3,2-d]pyrimidine-Based EGFR T790M/L858R Mutant Inhibitors. ACS Med. Chem. Lett..

[49] PDB of CA. https://www.rcsb.org/structure/3DC3.

[50] SwissADME. http://www.swissadme.ch/.

[51] Gundersen L.-L., Nissen-Meyer J., Spilsberg B. (2002). Synthesis and Antimycobacterial Activity of 6-Arylpurines: The Requirements for the N-9 Substituent in Active Antimycobacterial Purines. J. Med. Chem..

[52] Heifets L.B., Flory M.A., Lindholm-Levy P.J. (1989). Does pyrazinoic acid as an active moiety of pyrazinamide have specific activity against Mycobacterium tuberculosis?. Antimicrob. Agents Chemother..

[53] Refsgaard H.H.F., Jensen B.F., Brockhoff P.B., Padkjær S.B., Guldbrandt M., Christensen M.S. (2005). In Silico Prediction of Membrane Permeability from Calculated Molecular Parameters. J. Med. Chem..

[54] Veber D.F., Johnson S.R., Cheng H.-Y., Smith B.R., Ward K.W., Kopple K.D. (2002). Molecular Properties That Influence the Oral Bioavailability of Drug Candidates. J. Med. Chem..

[55] Lipinski C.A., Lombardo F., Dominy B.W., Feeney P.J. (2001). Experimental and computational approaches to estimate solubility and permeability in drug discovery and development settings. Adv. Drug Deliv. Rev..

[56] Baell J.B., Holloway G.A. (2010). New Substructure Filters for Removal of Pan Assay Interference Compounds (PAINS) from Screening Libraries and for Their Exclusion in Bioassays. J. Med. Chem..

[57] Bakonyi T., Radak Z. (2004). High Altitude and Free Radicals. J. Sports Sci. Med..

[58] Askew E. (2002). Work at high altitude and oxidative stress: Antioxidant nutrients. Toxicology.

[59] Forwand S.A., Landowne M., Follansbee J.N., Hansen J.E. (1968). Effect of Acetazolamide on Acute Mountain Sickness. N. Engl. J. Med..

[60] Shekhar S., Liu Y., Wang S., Zhang H., Fang X., Zhang J., Fan L., Zheng B., Roman R.J., Wang Z. (2021). Novel Mechanistic Insights and Potential Therapeutic Impact of TRPC6 in Neurovascular Coupling and Ischemic Stroke. Int. J. Mol. Sci..

