# Supplementary material for: Green Synthesis of Oxoquinoline-1(2H)-Carboxamide as Antiproliferative and Antioxidant Agents: An Experimental and In-Silico Approach to High Altitude Related Disorders
Source: Molecules. 2022 Jan 4;27(1):309. doi: 10.3390/molecules27010309 (PMC8746819; doi:10.3390/molecules27010309)
Supplement: Supplementary file 1 [file molecules-27-00309-s001.zip › molecules-1484839-supplementary.pdf]

Article

Green Synthesis of Oxoquinoline-1(2*H*)-Carboxamide as Antiproliferative and Antioxidant Agents: An Experimental and In-Silico Approach to High Altitude Related Disorders

Amena Ali [1,2,*], Abuzer Ali [3], Musarrat Husain Warsi [4], Mohammad Akhlaquer Rahman [4], Mohamed Jawed Ahsan [5] and Faizul Azam [6]

[1] High Altitude Research Center, Taif University, P.O. Box 11099, Taif 21944, Saudi Arabia; amrathore@tu.edu.sa
[2] Department of Pharmaceutical Chemistry, College of Pharmacy, Taif University, P.O. Box 11099, Taif 21944, Saudi Arabia; amrathore@tu.edu.sa
[3] Department of Pharmacognosy, College of Pharmacy, Taif University, P.O. Box 11099, Taif 21944, Saudi Arabia; abuali@tu.edu.sa
[4] Department of Pharmaceutics and Industrial Pharmacy, College of Pharmacy, Taif University, P.O. Box 11099, Taif 21944, Saudi Arabia; mvarsi@tu.edu.sa (M.H.W.); mrahman@tu.edu.sa (M.A.R.)
[5] Department of Pharmaceutical Chemistry, Maharishi Arvind College of Pharmacy, Ambabari Circle, Jaipur, Rajasthan 302 039, India; jawedpharma@gmail.com
[6] Department of Pharmaceutical Chemistry & Pharmacognosy, Unaizah College of Pharmacy, Qassim University, Uniazah 51911, Saudi Arabia; f.azam@qu.edu.sa
* Correspondence: amrathore@tu.edu.sa

Supplementary Information

Characterization of compounds 5a-j

***7-Hydroxy-4-methyl-2-oxo-N-phenylquinoline-1(2H)-carboxamide (5a)*:** ^{1}H NMR (300 MHz DMSO-*d*$_6$): δ 2.34 (3H, s, CH_3), 6.10 (1H, s, ArH, H8), 6.69 (1H, s, ArH, H3), 6.80 (1H, d, *J* = 6.1 Hz, ArH, H6), 6.85-6.87 (1H, m, *J* = 6.0 Hz, ArH, H4′), 7.17-7.22 (2H, m, ArH, H3′ & H5′), 7.37-7.43 (2H, m, ArH, H2′ & H6′), 7.58 (1H, d, *J* = 6.0 Hz, ArH, H5), 8.49 (1H, s, ArNH), 10.55 (1H, s, ArOH); ^{13}C NMR (75 MHz DMSO-*d*$_6$): δ 161.21, 160.36, 156.07, 154.88, 153.57, 140.55, 128.80, 126.63, 121.11, 118.24, 117.80, 112.91, 110.28, 102.22, 18.13; Anal. Calc. for $C_{17}H_{14}N_2O_3$: C, 69.38; H, 4.79; N, 9.52 found: C, 69.12; H, 4.75; N, 9.40%. EI-MS m/z = 2941 (M)$^+$,

***N-(4-Chlorophenyl)-7-hydroxy-4-methyl-2-oxoquinoline-1(2H)-carboxamide (5b)*:** ^{1}H NMR (300 MHz DMSO-*d*$_6$): δ 2.34 (3H, s, CH3), 6.11 (1H, s, ArH, H8), 6.69 (1H, s, ArH, H3), 6.80 (1H, d, *J* = 6.3 Hz, ArH, H6), 7.23 (2H, d, *J* = 6.0 Hz, ArH, H3′ & H5′), 7.39 (2H, d, *J* = 6.0 Hz, ArH, H2′ & H6′), 7.57 (1H, d, *J* = 6.3 Hz, ArH, H5), 8.30 (1H, s, ArNH), 10.49 (1H, s, ArOH); ^{13}C NMR (75 MHz DMSO-*d*$_6$): δ 161.15, 160.28, 153.50, 152.33, 139.80, 128.80, 126.58, 125.51, 124.51, 120.95, 119.82, 112.01, 110.25, 102.18, 18.09; Anal. Calc. for $C_{17}H_{13}ClN_2O_3$: C, 62.11; H, 3.99; N, 8.52 found: C, 62.05; H, 3.89; N, 8.40%. EI-MS m/z = 328.1 (M+H)$^+$, 330.1 (M+2)$^+$.

***7-Hydroxy-4-methyl-2-oxo-N-(p-tolyl)quinoline-1(2H)-carboxamide (5c)*:** ^{1}H NMR (300 MHz DMSO-*d*$_6$): δ 2.21 (3H, s, CH_3), 2.34 (3H, s, CH_3), 6.11 (1H, s, ArH, H8), 6.69 (1H, s, ArH, H3), 6.81 (1H, d, *J* = 6.1 Hz, ArH, H6), 7.10 (2H, d, *J* = 6.1 Hz, ArH, H3′ & H5′), 7.21 (1H, d, *J* = 6.1 Hz, ArH, H5), 7.34 (2H, d,

J = 6.0 Hz, ArH, H2′ & H6′), 8.71 (1H, s, ArNH), 10.51 (1H, s, ArOH); Anal. Calc. for $C_{18}H_{16}N_2O_3$: C, 70.12; H, 5.23; N, 9.09 found: C, 70.02; H, 5.17; N, 9.01%. EI-MS m/z = 308.1 $(M)^+$.

7-Hydroxy-N-(4-methoxyphenyl)-4-methyl-2-oxoquinoline-1(2H)-carboxamide (5d): ^{1}H NMR (300 MHz DMSO-d_6): δ 2.34 (3H, s, CH_3), 3.69 (3H, s, OCH_3), 6.09 (1H, s, ArH, H8), 6.68 (1H, s, ArH, H3), 6.76 (1H, d, *J* = 6.0 Hz, ArH, H6), 6.85 (2H, d, *J* = 6.1 Hz, ArH, H3′ & H5′), 7.25 (2H, d, *J* = 6.1 Hz, ArH, H2′ & H6′), 7.55 (1H, d, *J* = 6.0 Hz, ArH, H5), 8.31 (1H, s, ArNH), 10.50 (1H, s, ArOH); ^{13}C NMR (75 MHz DMSO-d_6): δ 161.21, 160.38, 156.35, 154.89, 153.55, 133.67, 132.99, 126.60, 120.02, 119.65, 114.00, 112.91, 110.31, 102.24, 55.15, 18.13; Anal. Calc. for $C_{18}H_{16}N_2O_3$: C, 66.66; H, 4.97; N, 8.64 found: C, 66.56; H, 4.90; N, 8.60%. EI-MS m/z = 324.1 $(M)^+$.

7-Hydroxy-4-methyl-2-oxo-N-(4-(trifluoromethyl)phenyl)quinoline-1(2H)-carboxamide (5e): ^{1}H NMR (300 MHz DMSO-d_6): δ 2.34 (3H, s, CH_3), 6.10 (1H, s, ArH, H8), 6.68 (1H, s, ArH, H3), 6.79 (1H, d, *J* = 6.1 Hz, ArH, H6), 7.27 (1H, d, *J* = 6.0 Hz, ArH, H5), 7.46 (2H, d, *J* = 6.3 Hz, ArH, H2′ & H6′), 7.57 (2H, d, J = 6.2 Hz, ArH, H3′ & H5′), 8.54 (1H, s, ArNH), 10.52 (1H, s, ArOH); Anal. Calc. for $C_{18}H_{13}F_3N_2O_3$: C, 59.67; H, 3.62; N, 7.73 found: C, 59.56; H, 3.60; N, 7.70%. EI-MS m/z = 362.1 $(M)^+$.

N-(2-Chlorophenyl)-7-hydroxy-4-methyl-2-oxoquinoline-1(2H)-carboxamide (5f): ^{1}H NMR (300 MHz DMSO-d_6): δ 2.34 (3H, s, CH_3), 6.10 (1H, s, ArH, H8), 6.68 (1H, s, ArH, H3), 6.79 (1H, d, *J* = 6.2 Hz, ArH, H6), 7.29-7.58 (4H, m, ArH), 8.01 (1H, d, *J* = 6.1 Hz, ArH, H8), 8.21 (1H, s, ArNH), 10.51 (1H, s, OH); ^{13}C NMR (75 MHz DMSO-*d6*): δ 161.17, 160.31, 154.85, 153.53, 151.73, 136.78, 135.18, 129.24, 127.69, 126.60, 124.05, 122.50, 121.33, 112.87, 110.27, 102.20, 18.11; Anal. Calc. for $C_{17}H_{13}ClN_2O_3$: C, 62.11; H, 3.99; N, 8.52 found: C, 62.08; H, 3.92; N, 8.45%. EI-MS m/z = 328.1 $(M+H)^+$, 330.1 $(M+2)^+$.

7-Hydroxy-4-methyl-2-oxo-N-(o-tolyl)quinoline-1(2H)-carboxamide (5g): ^{1}H NMR (300 MHz DMSO-d_6): δ 2.19 (3H, s, CH_3), 2.34 (3H, s, CH_3), 6.09 (1H, s, ArH, H8), 6.69 (1H, s, ArH, H3), 6.78 (1H, d, *J* = 6.0 Hz, ArH, H6), 7.09 (1H, d, *J* = 6.0 Hz, ArH), 7.12-7.32 (4H, m, ArH), 8.67 (1H, s, ArNH), 10.52 (1H, s, ArOH); Anal. Calc. for $C_{18}H_{16}N_2O_3$: C, 70.12; H, 5.23; N, 9.09 found: C, 70.08; H, 5.19; N, 9.02%. EI-MS m/z = 308.1 $(M)^+$.

7-Hydroxy-N-(2-methoxyphenyl)-4-methyl-2-oxoquinoline-1(2H)-carboxamide (5h): ^{1}H NMR (300 MHz DMSO-d_6): δ 2.34 (3H, s, CH_3), 3.84 (3H, s, OCH_3), 6.10 (1H, s, ArH, H8), 6.68 (1H, s, ArH, H3), 6.79 (1H, d, *J* = 6.1 Hz, ArH, H6), 7.12 (1H, d, *J* = 6.0 Hz, ArH, H5), 7.28-7.82 (4H, m, ArH), 8.57 (1H, s, ArNH), 10.52 (1H, s, ArOH); Anal. Calc. for $C_{18}H_{16}N_2O_3$: C, 66.66; H, 4.97; N, 8.64 found: C, 66.55; H, 4.91; N, 8.59%. EI-MS m/z = 324.1 $(M)^+$.

N-(5-Chloro-2-hydroxyphenyl)-7-hydroxy-4-mehyl-2-oxoquinoline-1(2H)-carboxamide (5i): ^{1}H NMR (300 MHz DMSO-d_6): δ 2.34 (3H, s, CH_3), 6.11 (1H, s, ArH, H8), 6.67 (1H, s, ArH, H3), 6.82 (1H, d, *J* = 6.1 Hz, ArH, H6), 6.89 (1H, d, *J* = 6.1 Hz, ArH, H3), 7.02 (1H, d, *J* = 6.0 Hz, ArH, H4), 7.09 (1H,d, *J* = 6.0 Hz, ArH, H5), 7.82 (1H, s, ArH, H6′), 8.68 (1H, s, ArNH), 10.12 (1H, s, ArOH), 10.53 (1H, s, ArOH); Anal. Calc. for $C_{17}H_{13}ClN_2O_3$: C, 59.23; H, 3.80; N, 8.13 found: C, 59.19; H, 3.75; N, 8.09%. EI-MS m/z = 344.1 $(M)^+$.

N-(3-Chloro-4-fluorophenyl)-7-hydroxy-4-methyl-2-oxoquinoline-1(2H)-carboxamide (5j): ^{1}H NMR (300 MHz DMSO-d_6): δ 2.35 (3H, s, CH3), 6.11 (1H, s, ArH, H3), 6.69 (1H, s, ArH, H8), 6.77 (2H, d, *J* = 6.1 Hz, ArH, H6 & H5′), 7.56 (2H, d, *J* = 6.0 Hz, ArH, H5 & H6′), 7.70 (1H, d, *J* = 6.1 Hz, ArH, H2′), 8.41 (1H, s, ArNH), 10.49 (1H, s, OH); ^{13}C NMR (75 MHz DMSO-d_6): δ 163.84, 161.13, 160.27, 154.82, 153,51, 150.52, 132.51, 126.59, 124.52, 122.81, 113.54, 112.84, 112.01, 110.23, 102.16, 18.11; Anal. Calc. for $C_{17}H_{12}ClFN_2O_3$: C, 58.89; H, 3.49; N, 5.48 found: C, 58.82; H, 3.45; N, 5.45%. EI-MS m/z = 346.1 $(M)^+$.

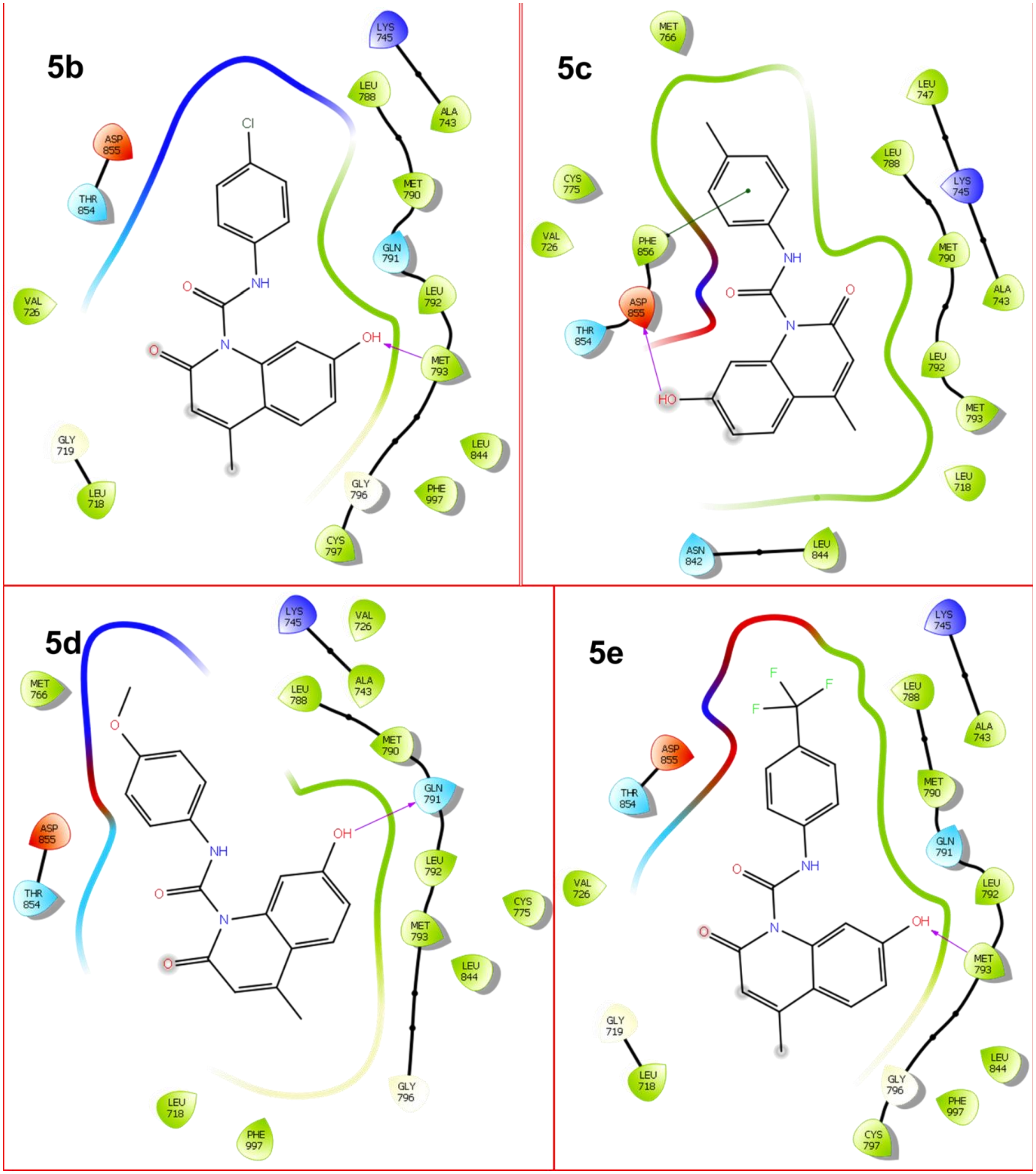
5b
Cl
NH
O
N
OH
ASP 855
THR 854
VAL 726
GLY 719
LEU 718
LYS 745
LEU 788
ALA 743
MET 790
GLN 791
LEU 792
MET 793
LEU 844
GLY 796
PHE 997
CYS 797
5c
MET 766
CYS 775
VAL 726
PHE 856
ASP 855
THR 854
HO
LEU 747
LEU 788
LYS 745
MET 790
ALA 743
LEU 792
MET 793
LEU 718
ASN 842
LEU 844
5d
MET 766
ASP 855
THR 854
LYS 745
VAL 726
LEU 788
ALA 743
MET 790
GLN 791
LEU 792
MET 793
CYS 775
LEU 844
GLY 796
LEU 718
PHE 997
5e
F
ASP 855
THR 854
VAL 726
GLY 719
LEU 718
LYS 745
LEU 788
ALA 743
MET 790
GLN 791
LEU 792
MET 793
LEU 844
GLY 796
PHE 997
CYS 797

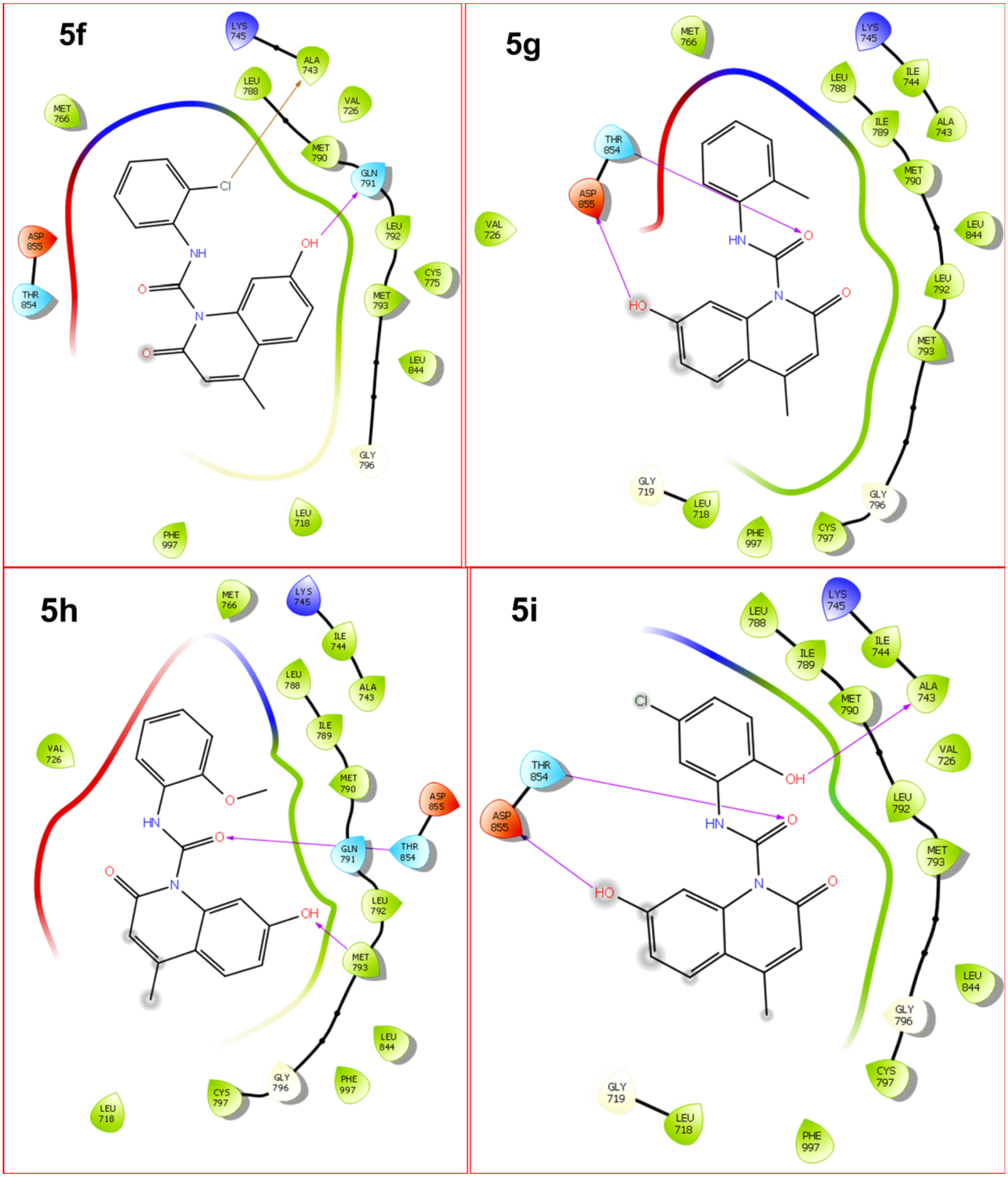

Figure 1S. The 2D interactions of ligands within the active site of EGFR

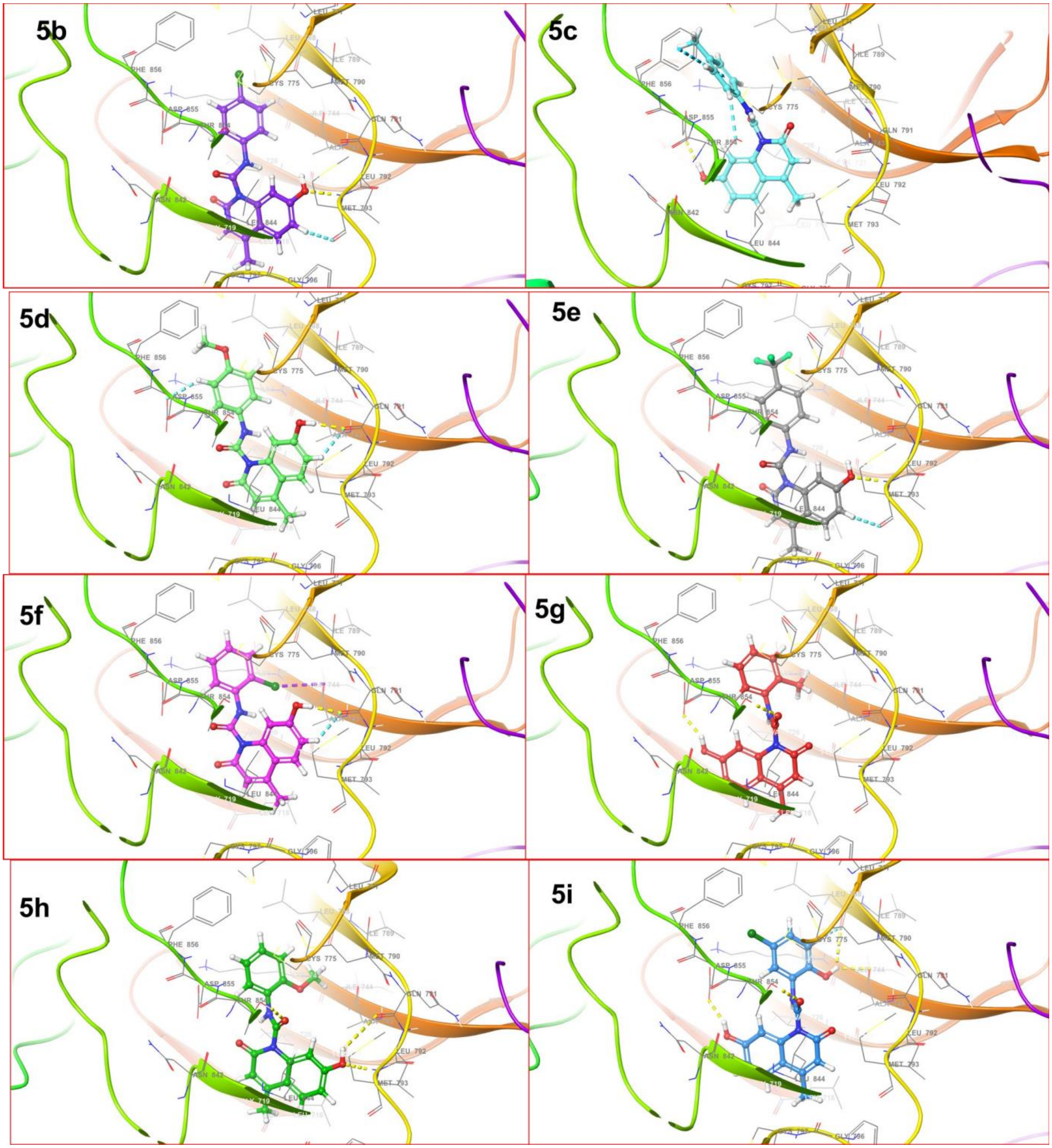

Figure 2S. The 3D interactions of ligands within the active site of EGFR.

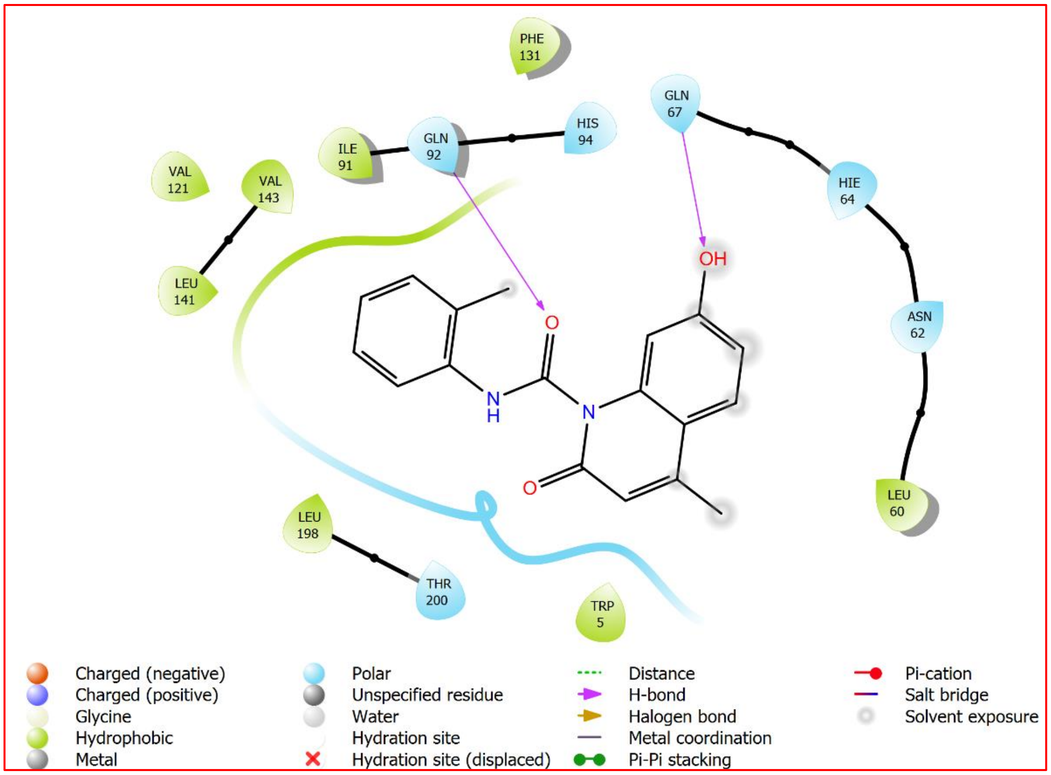

Figure 3S. The 3D interactions of ligand **5g** within the active site of CA IX.

Table 1S. The molecular docking results of compounds **5a-j** against CA IX (PDB ID: 3DC3).

S. No.	Ligand	Docking score	Types of interaction
1	**5a**	-5.337	H-bond (Gln67, Gln92, and Thr200)
2	**5b**	-4.175	H-bond (Thr200)
3	**5c**	-4.788	H-bond (Gln67 and Gln92)
4	**5d**	-4.078	H-bond (Gln92), π-π-Stacking (Phe131)
5	**5e**	-4.977	H-bond (Gln92), π-π-Stacking (Phe131)
6	**5f**	-4.384	H-bond (Gln67)
7	**5g**	-5.736	H-bond (Gln67 and Gln92)
8	**5h**	-5.643	H-bond (Gln67 and Gln92)
9	**5i**	-3.966	H-bond (Hie64), π-π-Stacking (Phe131)
10	**5j**	-4.291	H-bond (Gln92, Hie64, and Pro201), Halogen bond (Trp5 and Hip4)

Anticancer activity data of compounds 5a-j against 60 NCI cancer cell-lines

Developmental Therapeutics Program<br>One Dose Mean Graph	NSC: D-805519 / 1	Conc: 1.00E-5 Molar	Test Date: Jun 18, 2018
	Experiment ID: 1806OS76		Report Date: Jul 04, 2018

Panel/Cell Line	Growth Percent
Leukemia	
CCRF-CEM	105.80
HL-60(TB)	116.69
K-562	104.33
MOLT-4	113.67
RPMI-8226	105.43
SR	125.84
Non-Small Cell Lung Cancer	
A549/ATCC	95.94
EKVX	108.45
HOP-62	88.62
HOP-92	84.00
NCI-H226	100.62
NCI-H23	95.08
NCI-H322M	96.39
NCI-H460	96.53
NCI-H522	91.16
Colon Cancer	
COLO 205	106.00
HCC-2998	101.50
HCT-116	98.18
HCT-15	105.95
HT29	102.90
KM12	103.95
SW-620	94.41
CNS Cancer	
SF-268	96.13
SF-295	103.42
SF-539	95.12
SNB-19	102.71
SNB-75	90.68
U251	94.23
Melanoma	
LOX IMVI	98.46
MALME-3M	91.18
M14	96.33
MDA-MB-435	99.02
SK-MEL-2	102.38
SK-MEL-28	106.09
SK-MEL-5	102.15
UACC-257	94.80
UACC-62	98.88
Ovarian Cancer	
IGROV1	98.36
OVCAR-3	105.29
OVCAR-4	113.92
OVCAR-5	97.86
OVCAR-8	95.36
NCI/ADR-RES	103.20
SK-OV-3	97.61
Renal Cancer	
786-0	105.69
A498	102.40
ACHN	100.07
CAKI-1	87.61
RXF 393	105.95
SN12C	101.82
TK-10	17.02
UO-31	76.90
Prostate Cancer	
PC-3	95.90
DU-145	106.92
Breast Cancer	
MCF7	98.26
MDA-MB-231/ATCC	104.65
HS 578T	88.50
BT-549	101.73
T-47D	98.76
MDA-MB-468	108.58
Mean	98.76
Delta	81.74
Range	108.82

Mean Growth Percent - Growth Percent

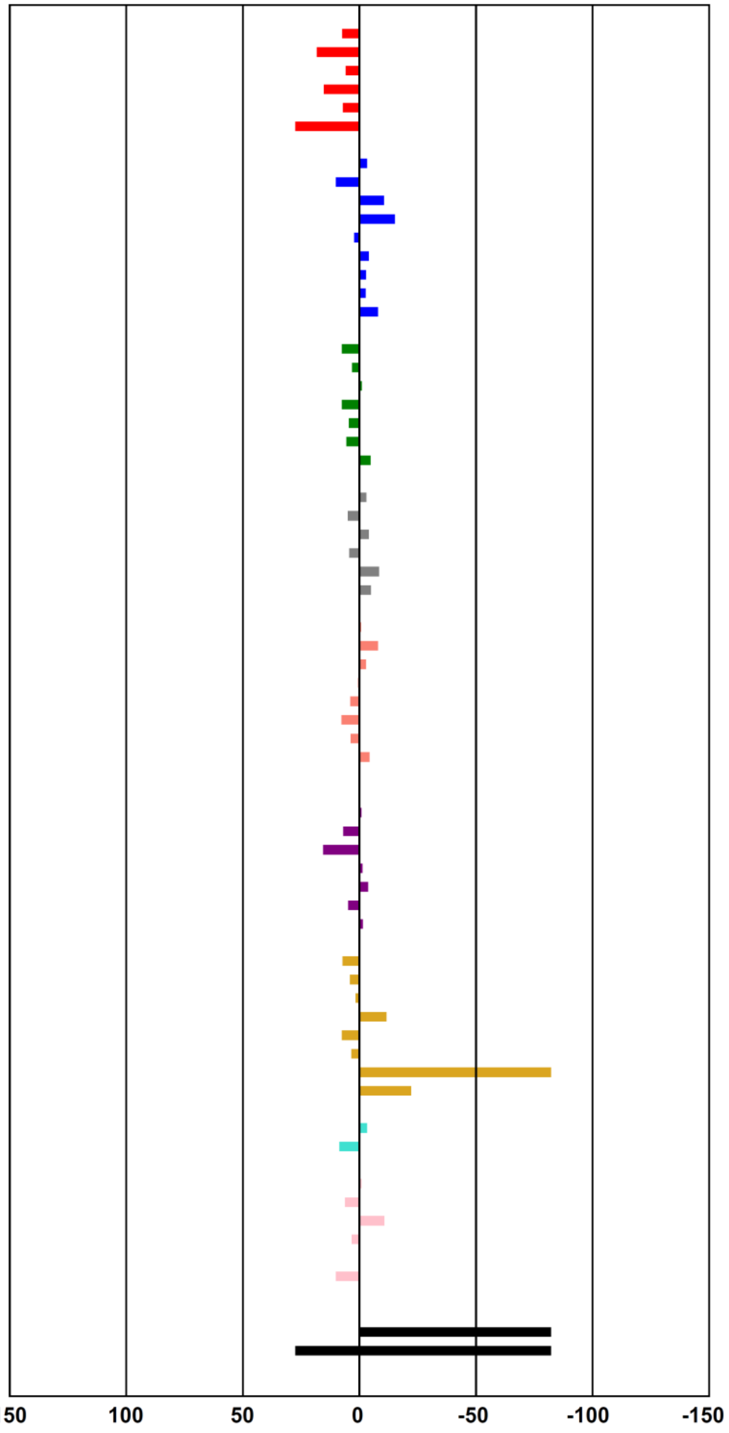

Developmental Therapeutics Program<br>One Dose Mean Graph	NSC: D-805508 / 1	Conc: 1.00E-5 Molar	Test Date: Jun 18, 2018
	Experiment ID: 1806OS76		Report Date: Jul 04, 2018

Panel/Cell Line	Growth Percent
**Leukemia**	
CCRF-CEM	102.37
HL-60(TB)	109.01
K-562	90.23
MOLT-4	100.47
RPMI-8226	104.09
SR	106.16
**Non-Small Cell Lung Cancer**	
A549/ATCC	102.33
EKVX	109.08
HOP-62	99.62
HOP-92	103.55
NCI-H226	103.81
NCI-H23	92.81
NCI-H322M	99.28
NCI-H460	110.14
NCI-H522	93.30
**Colon Cancer**	
COLO 205	113.88
HCC-2998	103.14
HCT-116	87.36
HCT-15	110.95
HT29	104.99
KM12	113.85
SW-620	103.45
**CNS Cancer**	
SF-268	97.41
SF-295	107.30
SF-539	100.70
SNB-19	102.49
SNB-75	91.49
U251	100.49
**Melanoma**	
LOX IMVI	97.57
MALME-3M	104.29
M14	104.60
MDA-MB-435	99.36
SK-MEL-2	110.49
SK-MEL-28	115.77
SK-MEL-5	99.15
UACC-257	102.33
UACC-62	95.20
**Ovarian Cancer**	
IGROV1	113.11
OVCAR-3	107.63
OVCAR-4	101.85
OVCAR-5	108.98
OVCAR-8	101.08
NCI/ADR-RES	105.17
SK-OV-3	97.72
**Renal Cancer**	
786-0	104.72
A498	100.91
ACHN	113.21
CAKI-1	96.48
RXF 393	104.95
SN12C	102.17
TK-10	115.49
UO-31	87.82
**Prostate Cancer**	
PC-3	100.69
DU-145	104.06
**Breast Cancer**	
MCF7	107.79
MDA-MB-231/ATCC	93.67
HS 578T	108.03
BT-549	103.77
T-47D	101.22
MDA-MB-468	97.19
Mean	102.84
Delta	15.48
Range	28.41

Mean Growth Percent - Growth Percent

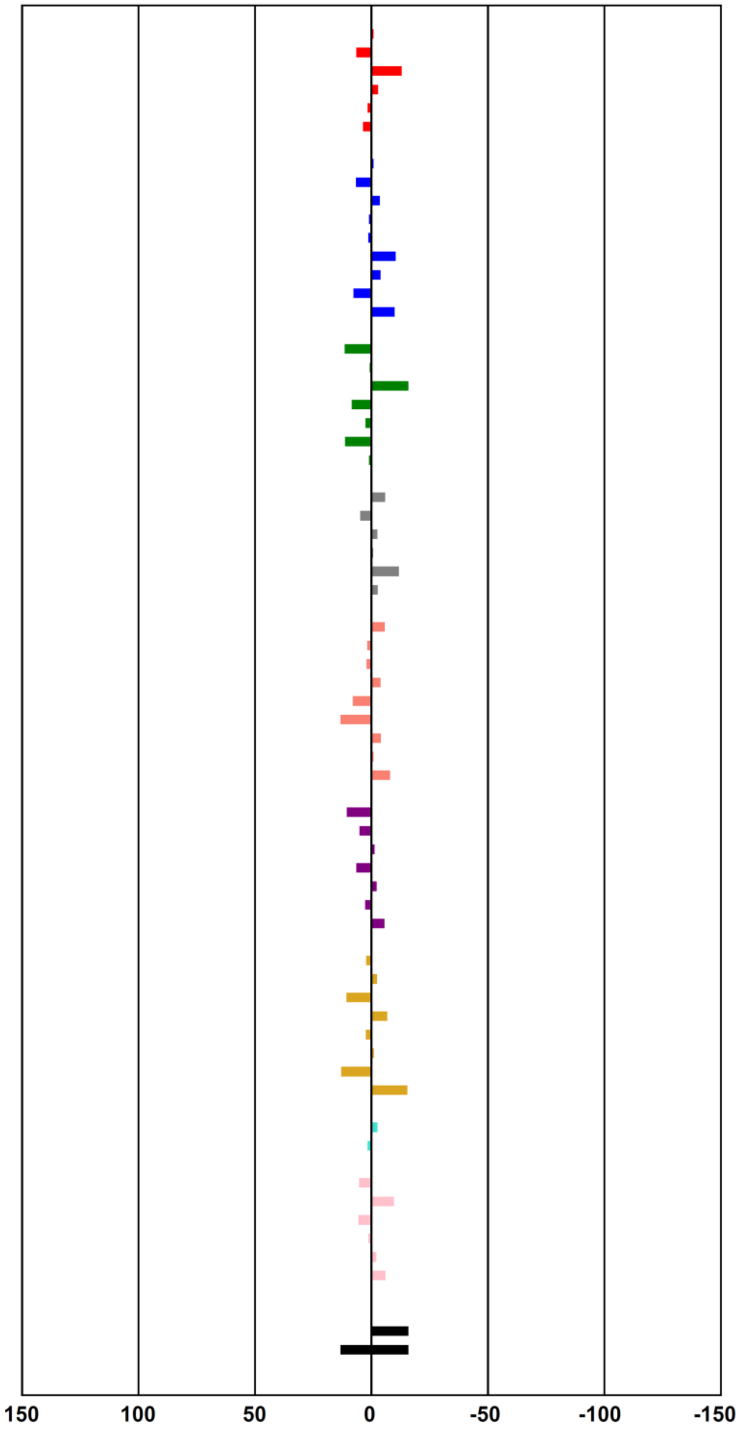

Developmental Therapeutics Program	NSC: D-805512 / 1	Conc: 1.00E-5 Molar	Test Date: Jun 18, 2018
One Dose Mean Graph	Experiment ID: 1806OS76		Report Date: Jul 04, 2018

Panel/Cell Line	Growth Percent
Leukemia	
CCRF-CEM	103.01
HL-60(TB)	102.93
K-562	116.68
MOLT-4	108.39
RPMI-8226	105.74
SR	110.38
Non-Small Cell Lung Cancer	
A549/ATCC	111.34
EKVX	92.99
HOP-62	101.55
HOP-92	85.60
NCI-H226	98.85
NCI-H23	102.23
NCI-H322M	93.63
NCI-H460	104.88
NCI-H522	103.64
Colon Cancer	
COLO 205	117.08
HCC-2998	108.56
HCT-116	99.12
HCT-15	104.89
HT29	115.09
KM12	110.58
SW-620	101.61
CNS Cancer	
SF-268	101.88
SF-295	102.53
SF-539	89.28
SNB-19	104.04
SNB-75	90.58
U251	107.89
Melanoma	
LOX IMVI	99.94
MALME-3M	90.95
M14	100.19
MDA-MB-435	109.63
SK-MEL-2	126.05
SK-MEL-28	101.00
SK-MEL-5	102.66
UACC-257	116.90
UACC-62	93.66
Ovarian Cancer	
IGROV1	99.24
OVCAR-3	116.69
OVCAR-4	113.71
OVCAR-5	87.13
OVCAR-8	101.29
NCI/ADR-RES	106.31
SK-OV-3	97.11
Renal Cancer	
786-0	104.94
A498	97.15
ACHN	100.23
CAKI-1	95.09
RXF 393	94.94
SN12C	94.93
TK-10	112.37
UO-31	82.04
Prostate Cancer	
PC-3	99.53
DU-145	114.99
Breast Cancer	
MCF7	107.70
MDA-MB-231/ATCC	95.23
HS 578T	99.57
BT-549	101.07
T-47D	100.50
MDA-MB-468	104.30
Mean	102.70
Delta	20.66
Range	44.01

Mean Growth Percent - Growth Percent

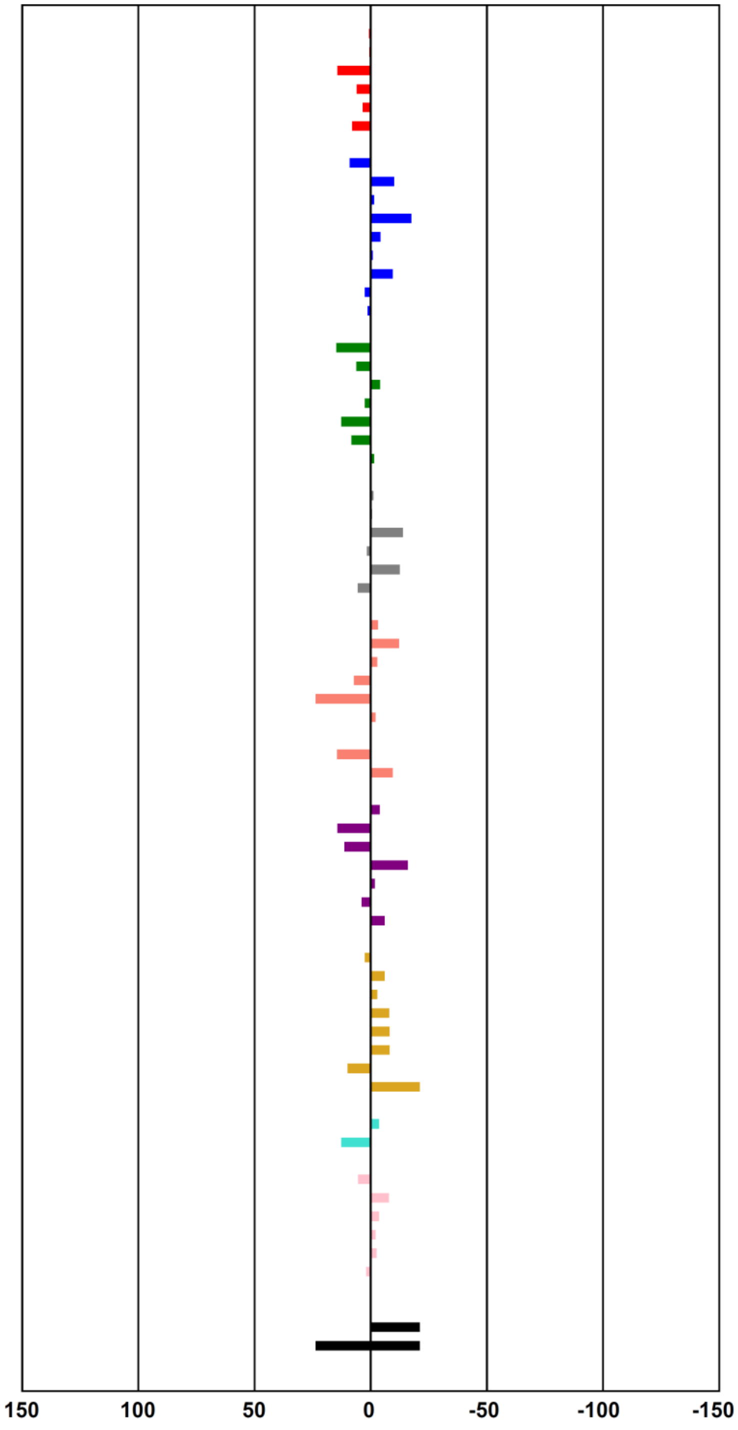

**Developmental Therapeutics Program**<br>**One Dose Mean Graph**	**NSC:** D-805516 / 1	**Conc:** 1.00E-5 Molar	**Test Date:** Jun 18, 2018
	**Experiment ID:** 1806OS76		**Report Date:** Jul 04, 2018

Panel/Cell Line	Growth Percent
Leukemia	
CCRF-CEM	92.39
HL-60(TB)	113.68
K-562	97.90
MOLT-4	102.67
RPMI-8226	96.86
SR	106.20
Non-Small Cell Lung Cancer	
A549/ATCC	106.83
EKVX	110.16
HOP-62	94.32
HOP-92	77.53
NCI-H226	93.24
NCI-H23	87.17
NCI-H322M	98.91
NCI-H460	108.60
NCI-H522	95.36
Colon Cancer	
COLO 205	117.16
HCC-2998	95.61
HCT-116	88.73
HCT-15	113.67
HT29	110.58
KM12	117.13
SW-620	97.72
CNS Cancer	
SF-268	95.66
SF-295	107.52
SF-539	98.84
SNB-19	99.48
SNB-75	86.10
U251	104.17
Melanoma	
LOX IMVI	92.00
MALME-3M	105.36
M14	105.17
MDA-MB-435	99.42
SK-MEL-2	113.68
SK-MEL-28	111.57
SK-MEL-5	103.66
UACC-257	108.09
UACC-62	89.65
Ovarian Cancer	
IGROV1	103.65
OVCAR-3	106.43
OVCAR-4	96.78
OVCAR-5	100.85
OVCAR-8	95.46
NCI/ADR-RES	103.01
SK-OV-3	90.97
Renal Cancer	
786-0	105.61
A498	106.57
ACHN	104.33
CAKI-1	89.65
RXF 393	111.29
SN12C	94.76
TK-10	130.97
UO-31	74.56
Prostate Cancer	
PC-3	95.88
DU-145	108.02
Breast Cancer	
MCF7	106.51
MDA-MB-231/ATCC	86.25
HS 578T	101.40
BT-549	102.12
T-47D	97.62
MDA-MB-468	100.78
Mean	100.94
Delta	26.38
Range	56.41

Mean Growth Percent - Growth Percent

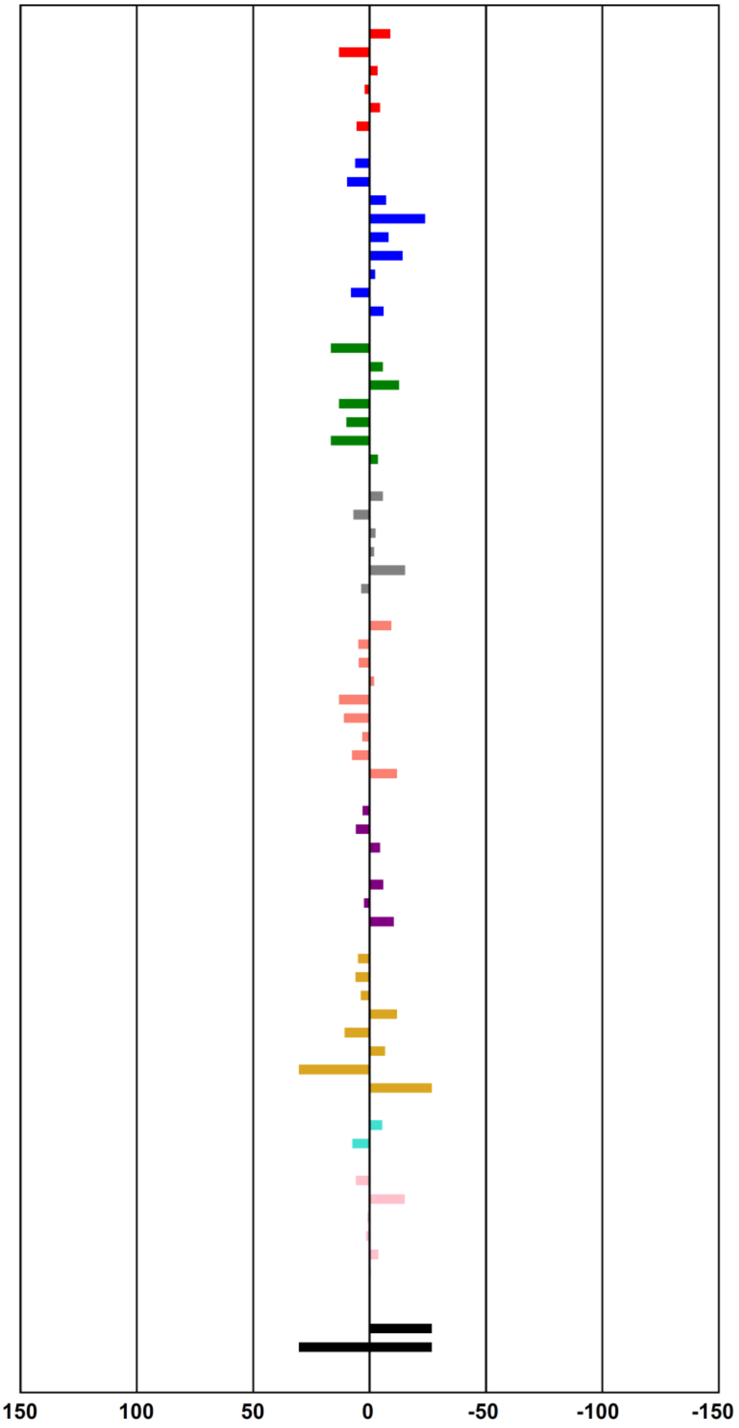

Developmental Therapeutics Program One Dose Mean Graph	NSC: D-805511 / 1	Conc: 1.00E-5 Molar	Test Date: Jun 18, 2018
	Experiment ID: 1806OS76		Report Date: Jul 04, 2018

Panel/Cell Line	Growth Percent
**Leukemia**	
CCRF-CEM	108.33
HL-60(TB)	116.63
K-562	101.88
MOLT-4	112.17
RPMI-8226	113.01
SR	122.29
**Non-Small Cell Lung Cancer**	
A549/ATCC	106.24
EKVX	107.15
HOP-62	96.19
HOP-92	95.97
NCI-H226	101.69
NCI-H23	95.95
NCI-H322M	99.03
NCI-H460	103.82
NCI-H522	98.62
**Colon Cancer**	
COLO 205	116.80
HCC-2998	104.82
HCT-116	103.43
HCT-15	105.12
HT29	111.03
KM12	108.87
SW-620	99.17
**CNS Cancer**	
SF-268	97.88
SF-295	100.68
SF-539	96.89
SNB-19	109.01
SNB-75	93.09
U251	105.28
**Melanoma**	
LOX IMVI	99.64
MALME-3M	106.50
M14	99.10
MDA-MB-435	102.76
SK-MEL-2	108.56
SK-MEL-28	104.74
SK-MEL-5	106.40
UACC-257	102.99
UACC-62	94.90
**Ovarian Cancer**	
IGROV1	107.16
OVCAR-3	102.95
OVCAR-4	105.75
OVCAR-5	98.94
OVCAR-8	97.83
NCI/ADR-RES	106.06
SK-OV-3	100.91
**Renal Cancer**	
786-0	111.07
A498	81.52
ACHN	103.73
CAKI-1	89.97
RXF 393	109.89
SN12C	98.07
TK-10	108.16
UO-31	87.76
**Prostate Cancer**	
PC-3	100.88
DU-145	105.34
**Breast Cancer**	
MCF7	101.57
MDA-MB-231/ATCC	100.09
HS 578T	96.83
BT-549	104.52
T-47D	101.12
MDA-MB-468	111.34
Mean	103.14
Delta	21.62
Range	40.77

Mean Growth Percent - Growth Percent

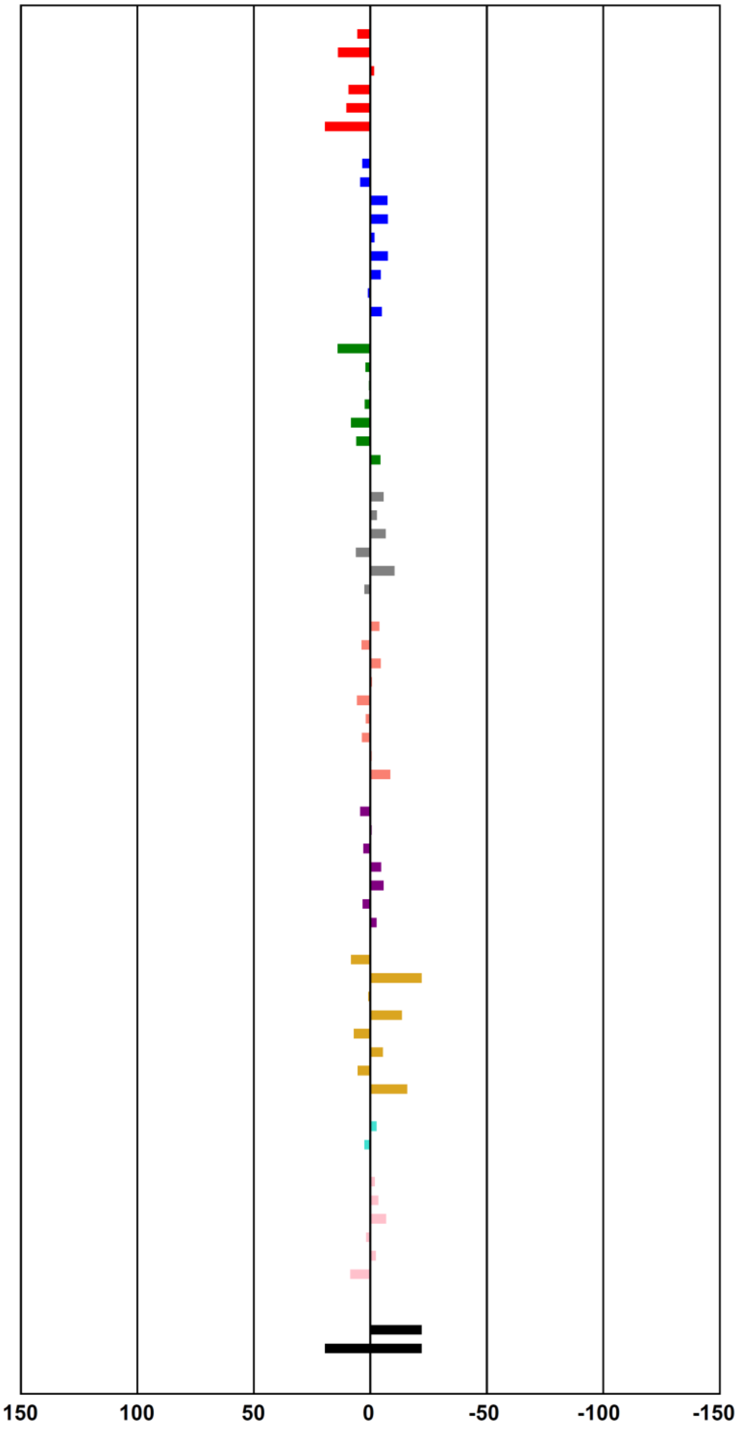

**Developmental Therapeutics Program**	**NSC:** D-805517 / 1	**Conc:** 1.00E-5 Molar	**Test Date:** Jun 18, 2018
**One Dose Mean Graph**	**Experiment ID:** 1806OS76		**Report Date:** Jul 04, 2018

Panel/Cell Line	Growth Percent
Leukemia	
CCRF-CEM	93.89
HL-60(TB)	107.31
K-562	109.00
MOLT-4	110.09
RPMI-8226	97.81
SR	112.42
Non-Small Cell Lung Cancer	
A549/ATCC	101.24
EKVX	108.42
HOP-62	89.21
HOP-92	79.74
NCI-H226	98.68
NCI-H23	98.64
NCI-H322M	88.75
NCI-H460	100.33
NCI-H522	95.23
Colon Cancer	
COLO 205	100.03
HCC-2998	98.54
HCT-116	101.39
HCT-15	107.33
HT29	107.54
KM12	98.07
SW-620	93.70
CNS Cancer	
SF-268	95.76
SF-295	101.23
SF-539	97.84
SNB-19	96.95
SNB-75	93.73
U251	98.61
Melanoma	
LOX IMVI	99.12
MALME-3M	92.95
M14	96.37
MDA-MB-435	104.33
SK-MEL-2	117.72
SK-MEL-28	107.25
SK-MEL-5	100.64
UACC-257	96.54
UACC-62	101.00
Ovarian Cancer	
IGROV1	103.59
OVCAR-3	105.77
OVCAR-4	106.99
OVCAR-5	102.44
OVCAR-8	95.47
NCI/ADR-RES	99.94
SK-OV-3	95.05
Renal Cancer	
786-0	103.34
A498	109.01
ACHN	100.26
CAKI-1	92.27
RXF 393	109.35
SN12C	98.46
TK-10	100.76
UO-31	76.87
Prostate Cancer	
PC-3	93.60
DU-145	108.31
Breast Cancer	
MCF7	101.02
MDA-MB-231/ATCC	100.79
HS 578T	98.69
BT-549	94.44
T-47D	97.11
MDA-MB-468	104.20
Mean	99.92
Delta	23.05
Range	40.85

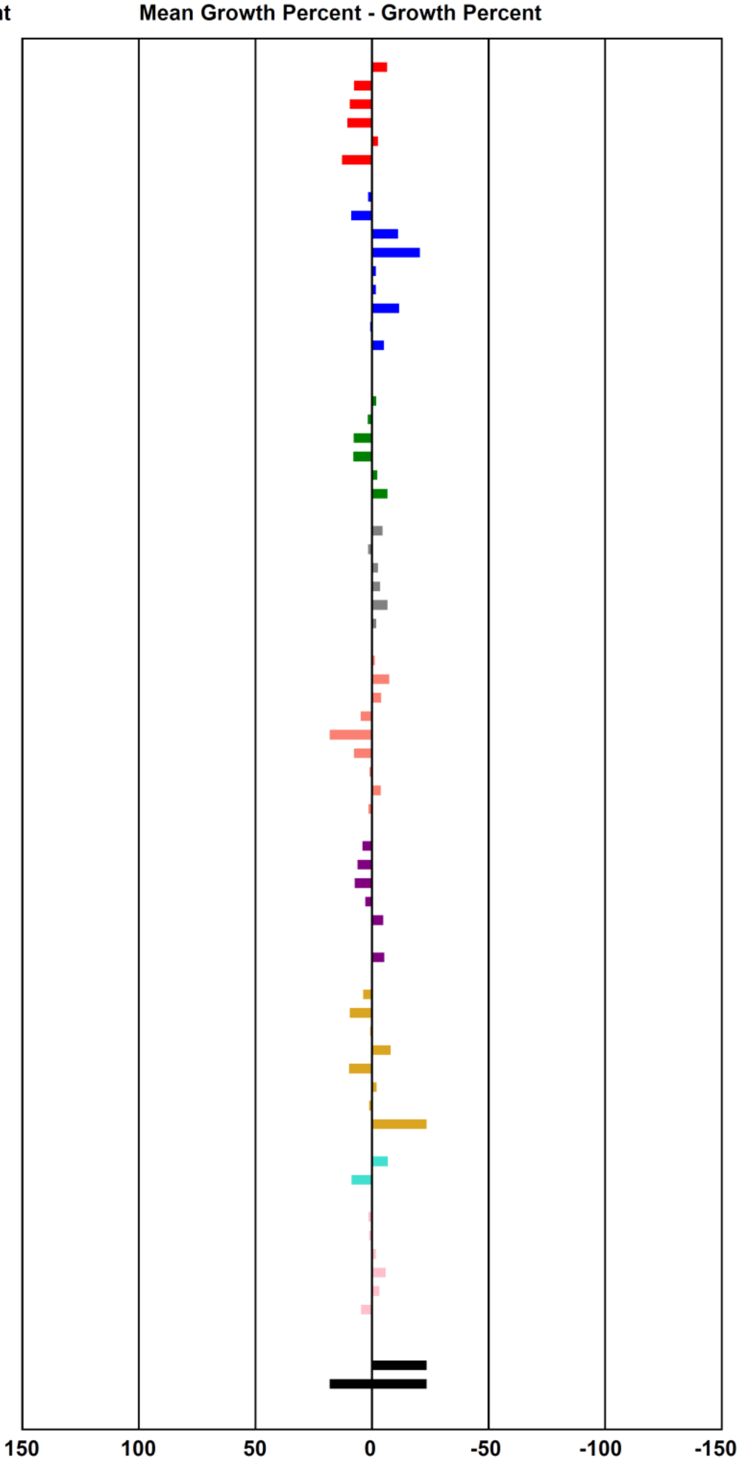

Developmental Therapeutics Program	NSC: D-805513 / 1	Conc: 1.00E-5 Molar	Test Date: Jun 18, 2018
One Dose Mean Graph	Experiment ID: 1806OS76		Report Date: Jul 04, 2018

Panel/Cell Line	Growth Percent
Leukemia	
CCRF-CEM	110.16
HL-60(TB)	104.82
K-562	113.77
MOLT-4	108.23
RPMI-8226	103.05
SR	124.16
Non-Small Cell Lung Cancer	
A549/ATCC	106.42
EKVX	107.08
HOP-62	99.32
HOP-92	89.43
NCI-H226	96.11
NCI-H23	106.82
NCI-H322M	102.69
NCI-H460	104.31
NCI-H522	100.66
Colon Cancer	
COLO 205	118.41
HCC-2998	111.10
HCT-116	101.64
HCT-15	108.22
HT29	115.19
KM12	105.84
SW-620	103.51
CNS Cancer	
SF-268	97.71
SF-295	99.16
SF-539	94.71
SNB-19	105.50
SNB-75	94.53
U251	100.16
Melanoma	
LOX IMVI	98.38
MALME-3M	102.35
M14	109.66
MDA-MB-435	107.98
SK-MEL-2	121.63
SK-MEL-28	110.87
SK-MEL-5	105.25
UACC-257	108.39
UACC-62	98.54
Ovarian Cancer	
IGROV1	108.92
OVCAR-3	106.14
OVCAR-4	110.44
OVCAR-5	99.13
OVCAR-8	101.63
NCI/ADR-RES	105.99
SK-OV-3	103.29
Renal Cancer	
786-0	108.39
A498	110.24
ACHN	105.09
CAKI-1	92.87
RXF 393	112.88
SN12C	95.96
TK-10	119.52
UO-31	80.06
Prostate Cancer	
PC-3	100.98
DU-145	112.52
Breast Cancer	
MCF7	109.06
MDA-MB-231/ATCC	90.02
HS 578T	94.23
BT-549	109.92
T-47D	107.01
MDA-MB-468	104.92
Mean	104.75
Delta	24.69
Range	44.10

Mean Growth Percent - Growth Percent

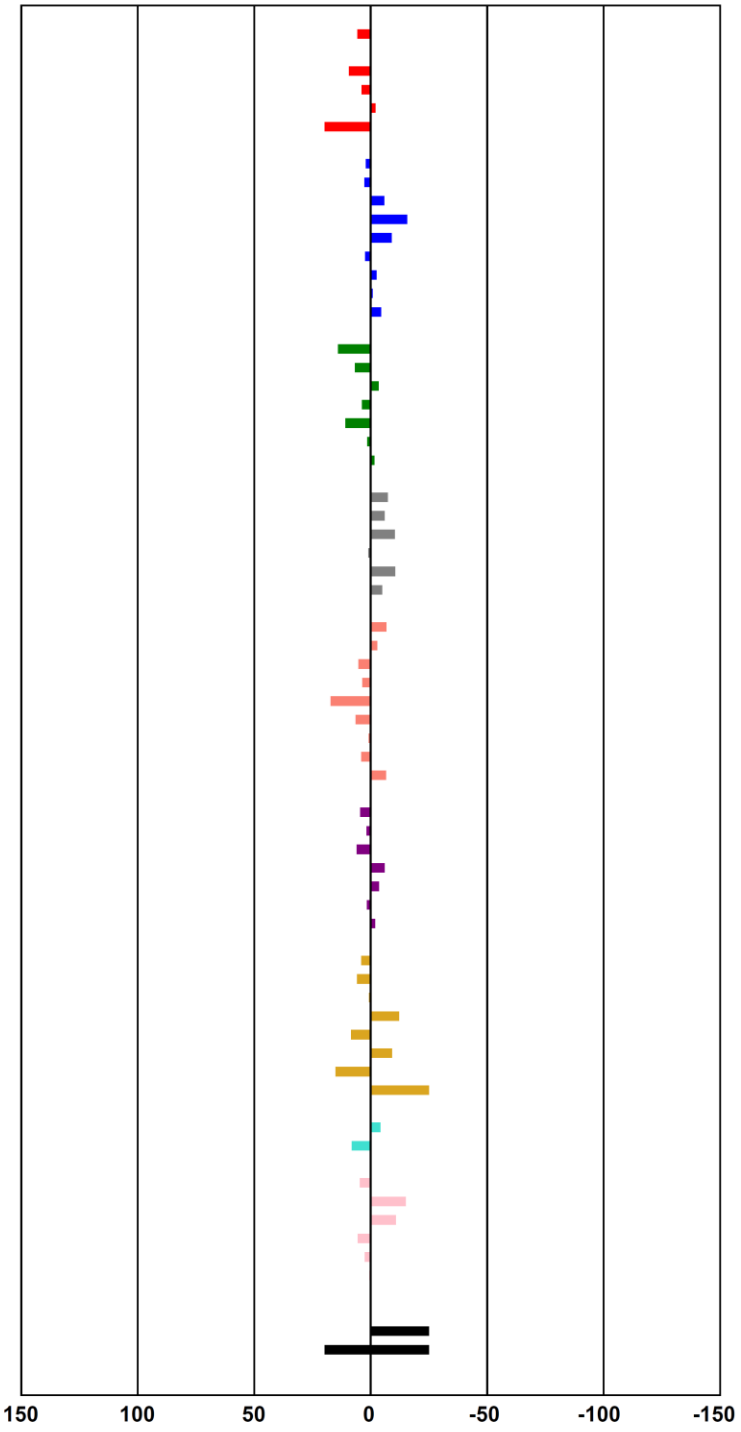

Developmental Therapeutics Program One Dose Mean Graph	NSC: D-805515 / 1	Conc: 1.00E-5 Molar	Test Date: Jun 18, 2018
	Experiment ID: 1806OS76		Report Date: Jul 04, 2018

Panel/Cell Line	Growth Percent
**Leukemia**	
CCRF-CEM	107.03
HL-60(TB)	105.34
K-562	116.78
MOLT-4	103.48
RPMI-8226	109.39
SR	117.68
**Non-Small Cell Lung Cancer**	
A549/ATCC	104.67
EKVX	100.45
HOP-62	102.28
HOP-92	88.90
NCI-H226	93.46
NCI-H23	101.57
NCI-H322M	103.22
NCI-H460	111.11
NCI-H522	96.16
**Colon Cancer**	
COLO 205	109.55
HCC-2998	95.92
HCT-116	106.62
HCT-15	104.14
HT29	106.21
KM12	101.26
SW-620	108.65
**CNS Cancer**	
SF-268	105.94
SF-295	101.22
SF-539	106.46
SNB-19	106.37
SNB-75	100.66
U251	105.71
**Melanoma**	
LOX IMVI	104.89
MALME-3M	105.88
M14	113.03
MDA-MB-435	104.72
SK-MEL-2	100.59
SK-MEL-28	113.34
SK-MEL-5	98.29
UACC-257	111.24
UACC-62	100.07
**Ovarian Cancer**	
IGROV1	112.62
OVCAR-3	113.26
OVCAR-4	110.04
OVCAR-5	112.18
OVCAR-8	105.87
NCI/ADR-RES	107.32
SK-OV-3	100.39
**Renal Cancer**	
786-0	114.93
A498	112.59
ACHN	109.67
CAKI-1	99.64
RXF 393	94.83
SN12C	100.83
TK-10	111.18
UO-31	88.62
**Prostate Cancer**	
PC-3	99.87
DU-145	112.57
**Breast Cancer**	
MCF7	100.84
MDA-MB-231/ATCC	106.12
HS 578T	109.98
BT-549	106.94
T-47D	99.76
MDA-MB-468	96.28
Mean	104.98
Delta	16.36
Range	29.06

Mean Growth Percent - Growth Percent

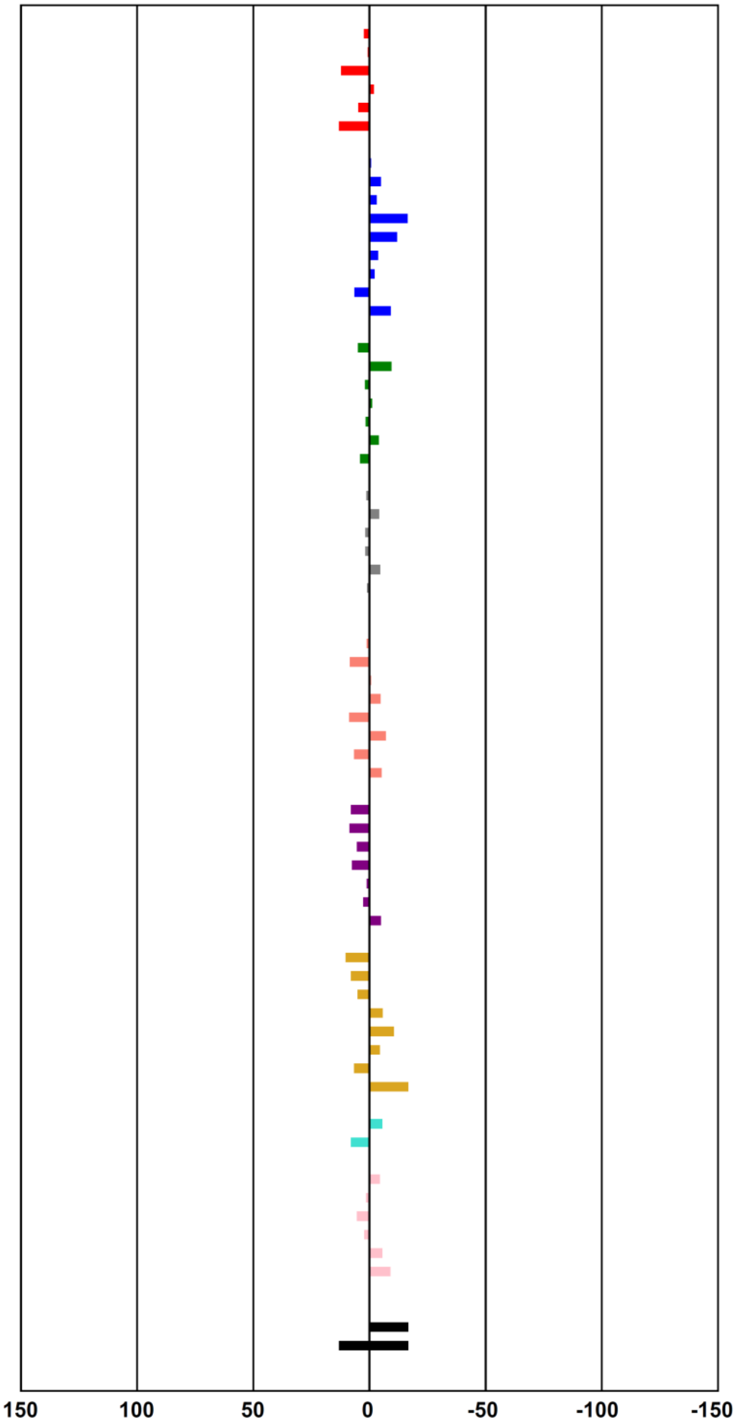

**Developmental Therapeutics Program**	**NSC:** D-805520 / 1	**Conc:** 1.00E-5 Molar	**Test Date:** Jun 18, 2018
**One Dose Mean Graph**	**Experiment ID:** 1806OS76		**Report Date:** Jul 04, 2018

Panel/Cell Line	Growth Percent
Leukemia	
CCRF-CEM	80.64
HL-60(TB)	99.94
K-562	110.39
MOLT-4	105.77
RPMI-8226	93.94
SR	108.78
Non-Small Cell Lung Cancer	
A549/ATCC	102.88
EKVX	103.00
HOP-62	94.99
HOP-92	72.79
NCI-H226	95.31
NCI-H23	97.23
NCI-H322M	88.16
NCI-H460	101.52
NCI-H522	94.33
Colon Cancer	
COLO 205	106.74
HCC-2998	109.42
HCT-116	96.09
HCT-15	99.09
HT29	99.97
KM12	104.96
SW-620	97.23
CNS Cancer	
SF-268	104.62
SF-295	104.66
SF-539	89.67
SNB-19	101.02
SNB-75	96.77
U251	103.49
Melanoma	
LOX IMVI	101.96
MALME-3M	87.21
M14	96.09
MDA-MB-435	105.94
SK-MEL-2	109.89
SK-MEL-28	101.36
SK-MEL-5	102.28
UACC-257	99.90
UACC-62	96.78
Ovarian Cancer	
IGROV1	98.42
OVCAR-3	117.95
OVCAR-4	121.90
OVCAR-5	99.51
OVCAR-8	94.27
NCI/ADR-RES	105.65
SK-OV-3	90.70
Renal Cancer	
786-0	103.22
A498	100.26
ACHN	99.29
CAKI-1	98.56
RXF 393	93.93
SN12C	95.87
TK-10	112.01
UO-31	79.09
Prostate Cancer	
PC-3	92.57
DU-145	111.40
Breast Cancer	
MCF7	99.47
MDA-MB-231/ATCC	92.13
HS 578T	95.38
BT-549	102.07
T-47D	96.63
MDA-MB-468	94.22
Mean	99.32
Delta	26.53
Range	49.11

Mean Growth Percent - Growth Percent

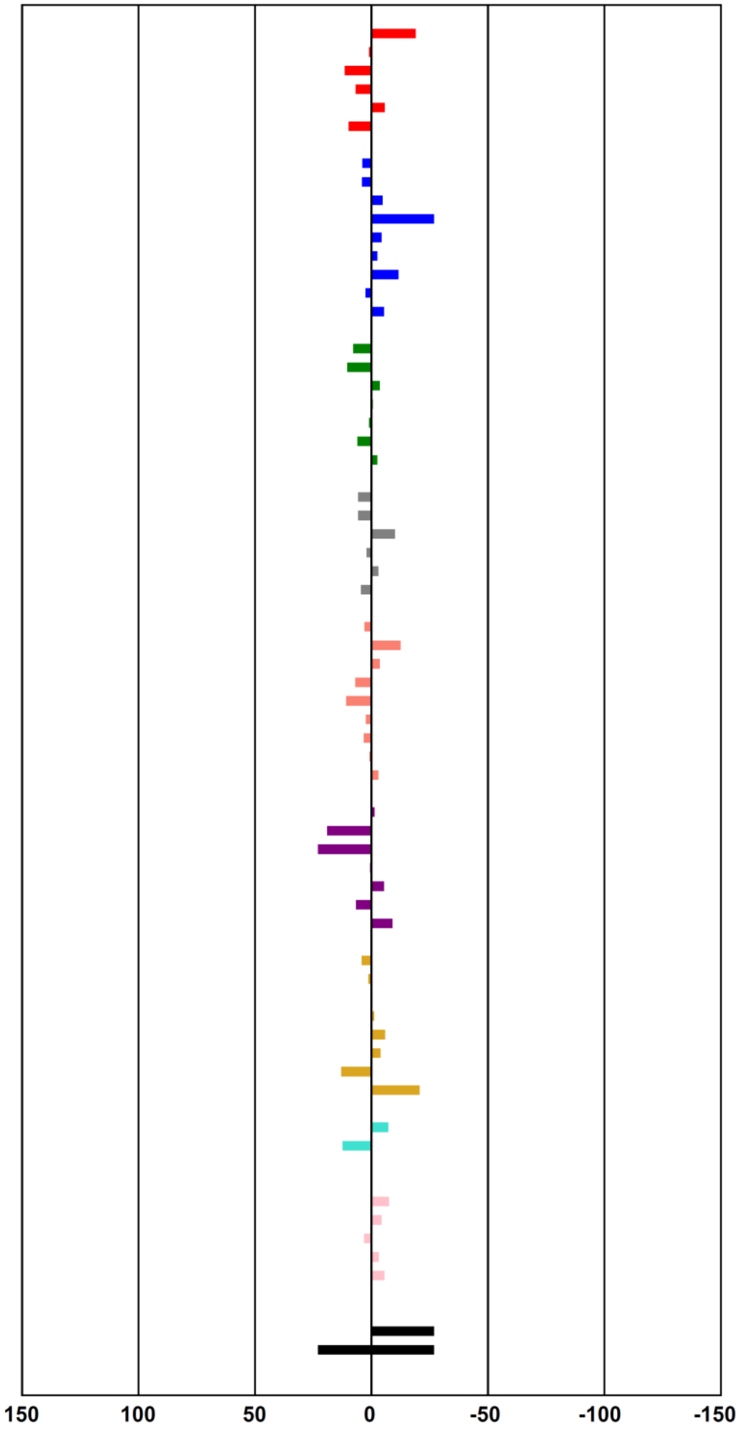

**Developmental Therapeutics Program**	**NSC:** D-805518 / 1	**Conc:** 1.00E-5 Molar	**Test Date:** Jun 18, 2018
**One Dose Mean Graph**	**Experiment ID:** 1806OS76		**Report Date:** Jul 04, 2018

Panel/Cell Line	Growth Percent
Leukemia	
CCRF-CEM	41.39
HL-60(TB)	113.17
K-562	120.89
MOLT-4	108.63
RPMI-8226	98.81
SR	117.33
Non-Small Cell Lung Cancer	
A549/ATCC	93.63
EKVX	104.14
HOP-62	82.44
HOP-92	72.56
NCI-H226	103.19
NCI-H23	92.86
NCI-H322M	94.62
NCI-H460	99.03
NCI-H522	88.23
Colon Cancer	
COLO 205	94.56
HCC-2998	101.45
HCT-116	88.16
HCT-15	107.58
HT29	101.30
KM12	99.70
SW-620	95.34
CNS Cancer	
SF-268	95.95
SF-295	103.69
SF-539	98.21
SNB-19	102.53
SNB-75	93.87
U251	93.40
Melanoma	
LOX IMVI	100.34
MALME-3M	94.32
M14	96.56
MDA-MB-435	105.63
SK-MEL-2	100.24
SK-MEL-28	106.62
SK-MEL-5	110.03
UACC-257	96.56
UACC-62	103.42
Ovarian Cancer	
IGROV1	104.25
OVCAR-3	104.64
OVCAR-4	107.26
OVCAR-5	94.07
OVCAR-8	92.22
NCI/ADR-RES	99.40
SK-OV-3	88.79
Renal Cancer	
786-0	96.74
A498	79.47
ACHN	100.47
CAKI-1	89.31
RXF 393	116.42
SN12C	106.36
TK-10	111.10
UO-31	70.00
Prostate Cancer	
PC-3	95.59
DU-145	108.72
Breast Cancer	
MCF7	101.16
MDA-MB-231/ATCC	106.86
HS 578T	92.85
BT-549	91.79
T-47D	95.99
MDA-MB-468	122.59
Mean	98.27
Delta	56.88
Range	81.20

Mean Growth Percent - Growth Percent

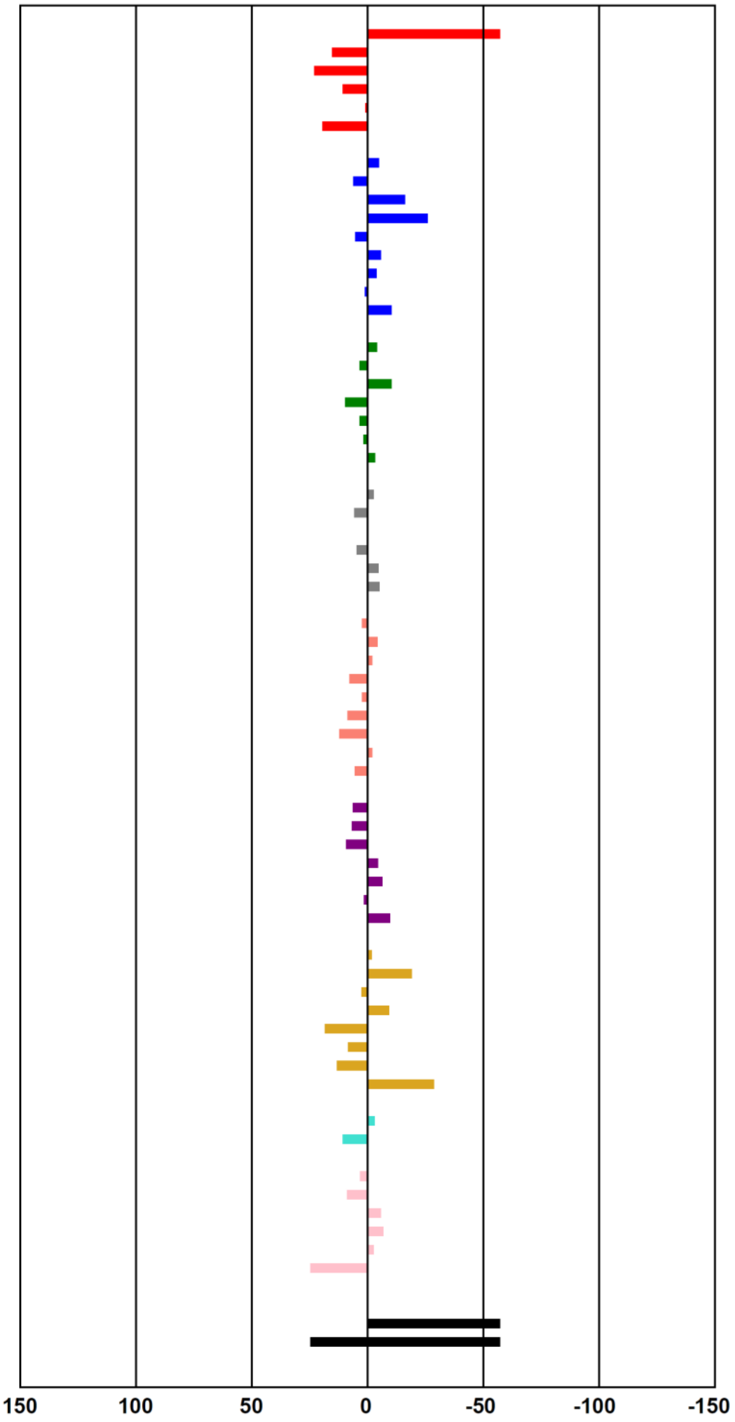

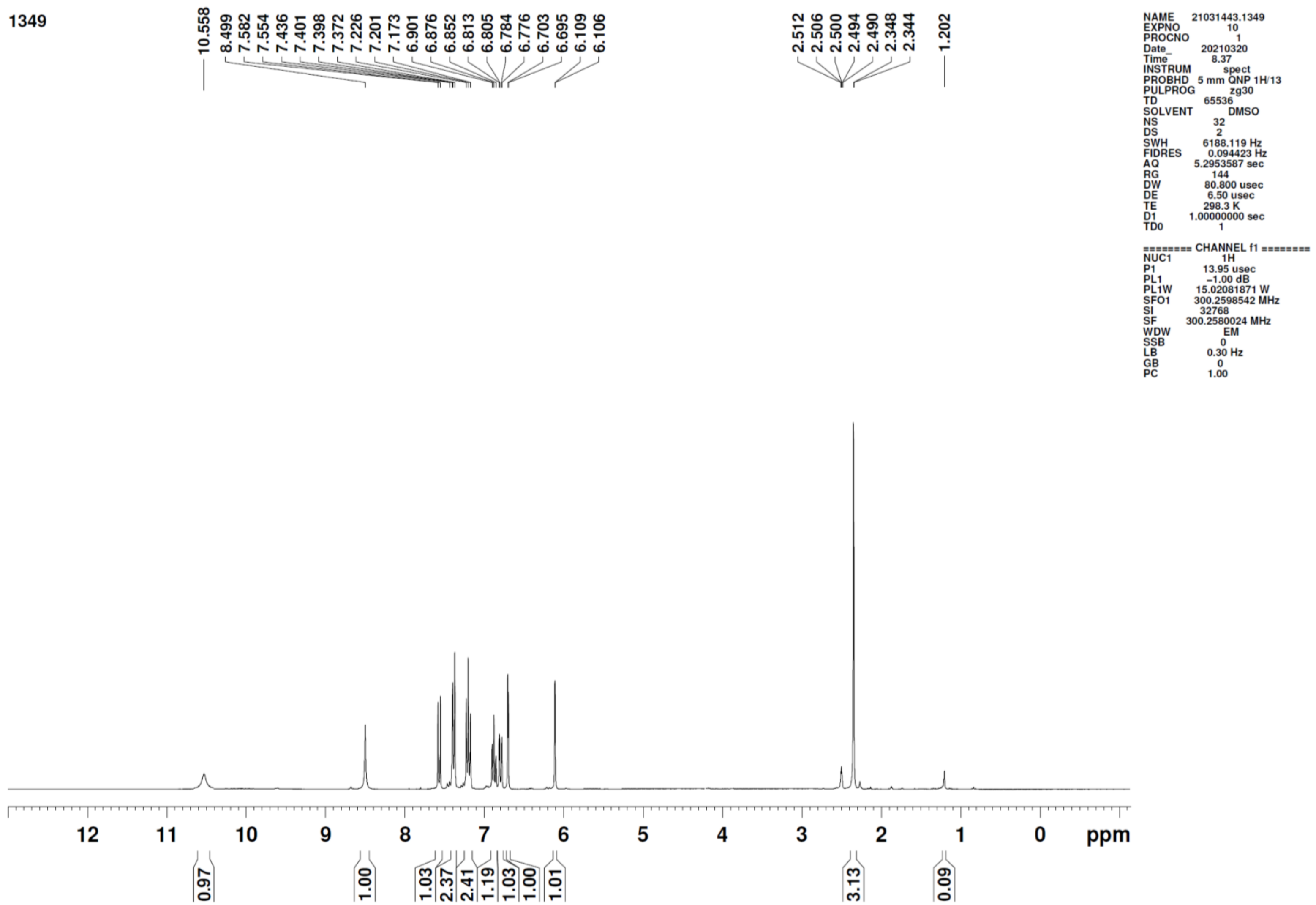

Spectra of compound 7-Hydroxy-4-methyl-2-oxo-*N*-phenylquinoline-1(2*H*)-carboxamide (**5a**)

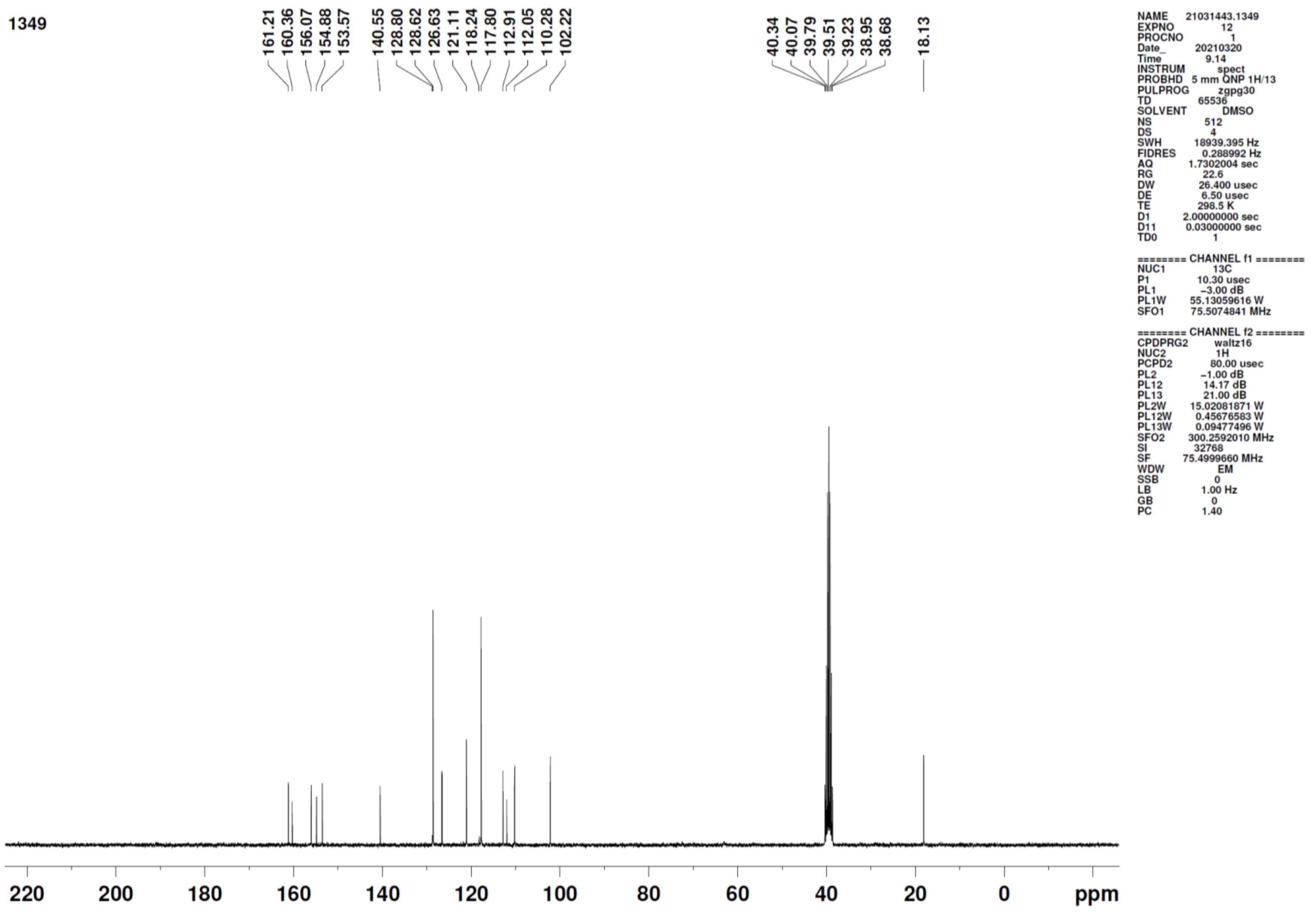

Spectra of compound 7-Hydroxy-4-methyl-2-oxo-*N*-phenylquinoline-1(2*H*)-carboxamide (**5a**)

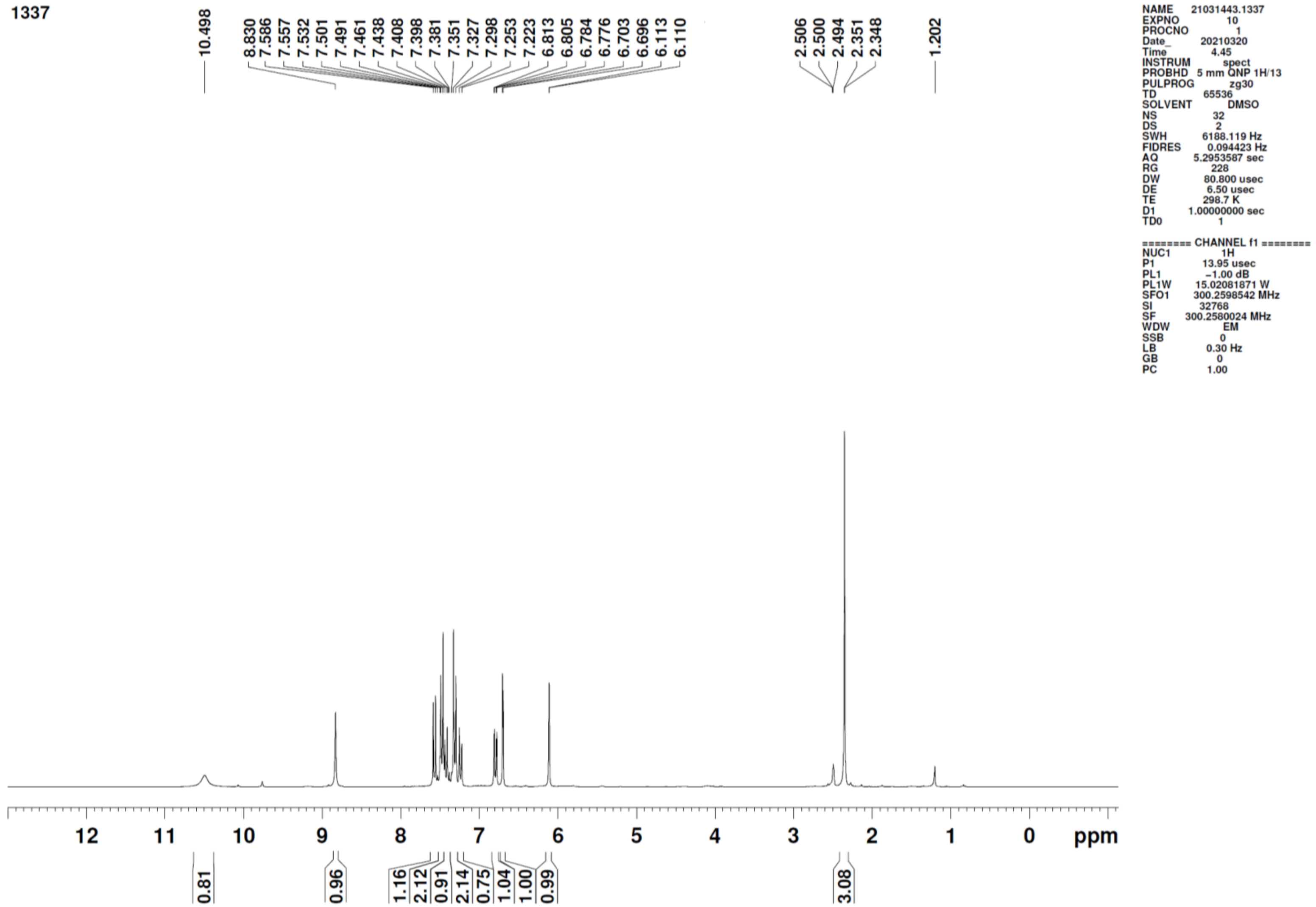

Spectra of compound *N*-(4-Chlorophenyl)-7-hydroxy-4-methyl-2-oxoquinoline-1(2*H*)-carboxamide (**5b**)

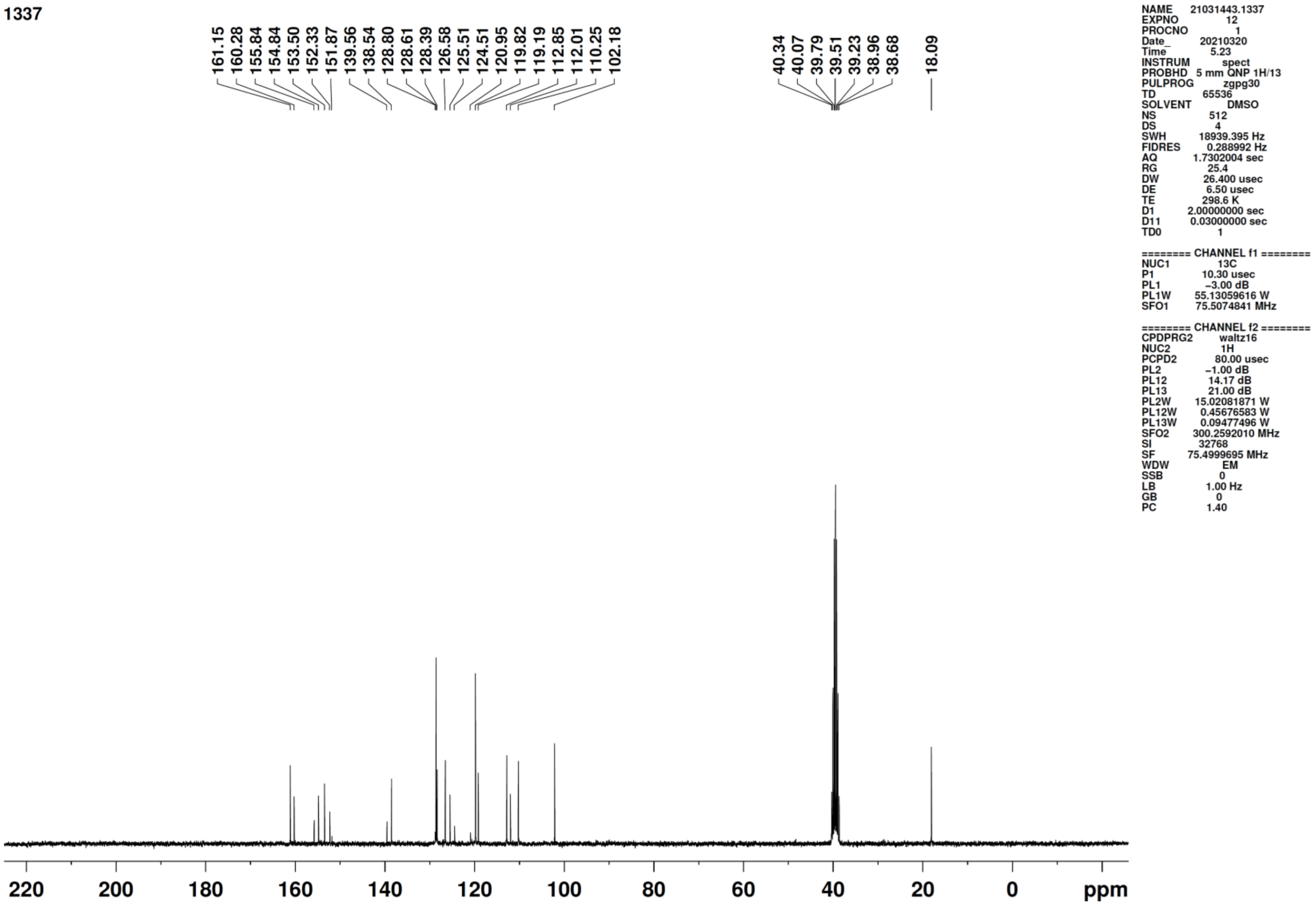

Spectra of compound *N*-(4-Chlorophenyl)-7-hydroxy-4-methyl-2-oxoquinoline-1(2*H*)-carboxamide (**5b**)

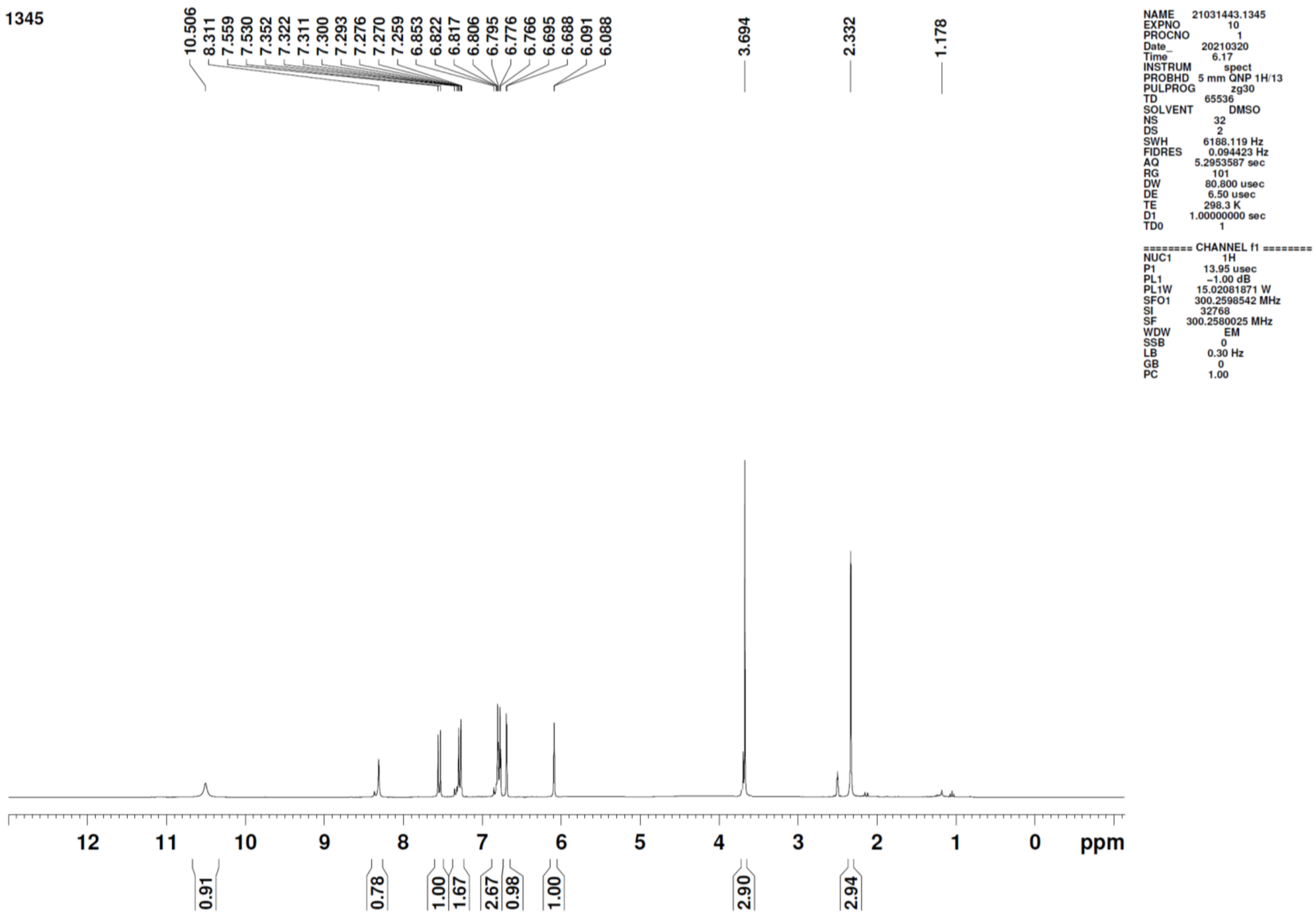

Spectra of 7-Hydroxy-*N*-(4-methoxyphenyl)-4-methyl-2-oxoquinoline-1(2*H*)-carboxamide (**5b**)

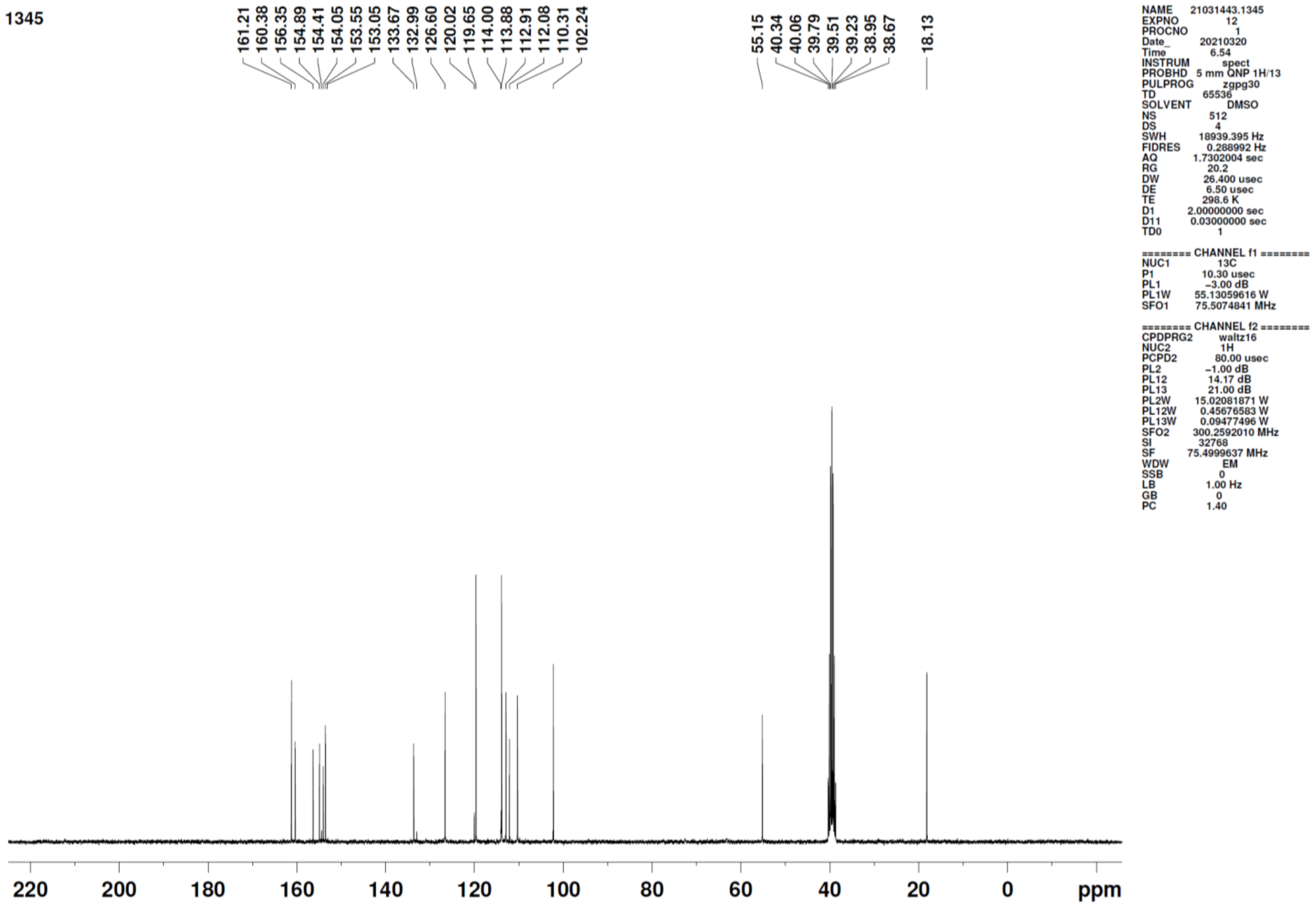

Spectra of 7-Hydroxy-*N*-(4-methoxyphenyl)-4-methyl-2-oxoquinoline-1(2*H*)-carboxamide (**5b**)

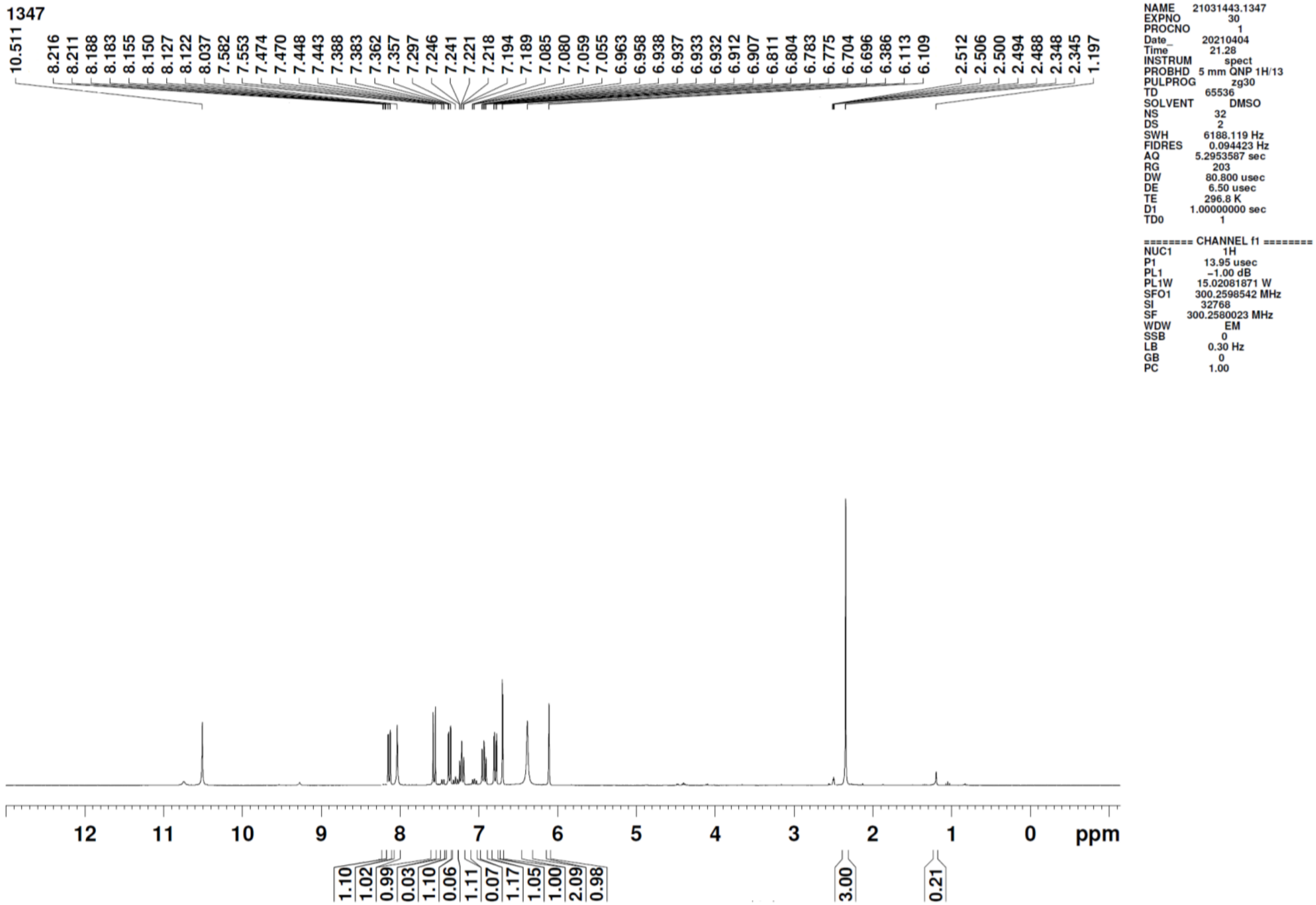

Spectra of *N*-(2-Chlorophenyl)-7-hydroxy-4-methyl-2-oxoquinoline-1(2*H*)-carboxamide (**5f**)

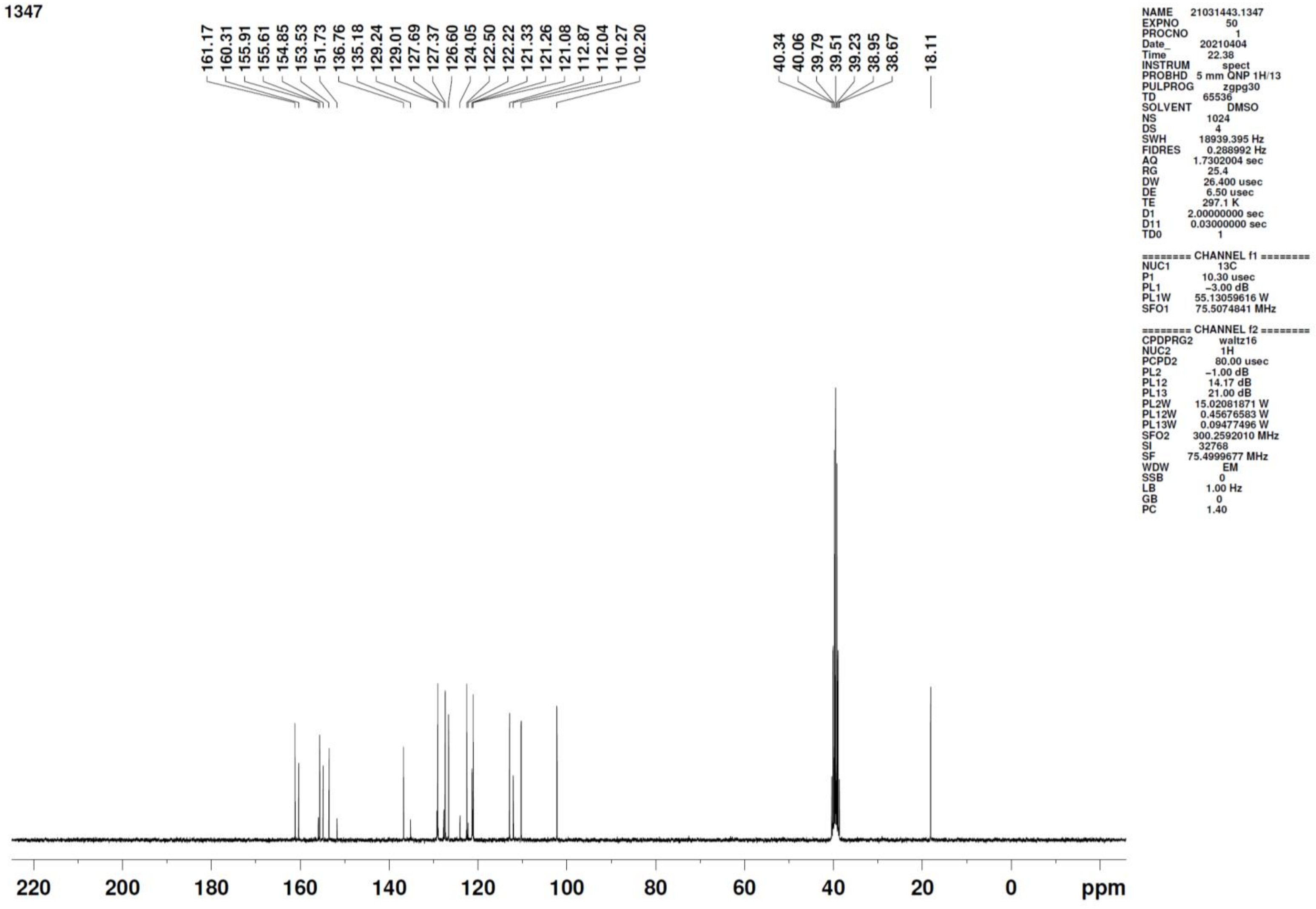

Spectra of *N*-(2-Chlorophenyl)-7-hydroxy-4-methyl-2-oxoquinoline-1(2*H*)-carboxamide (**5f**)

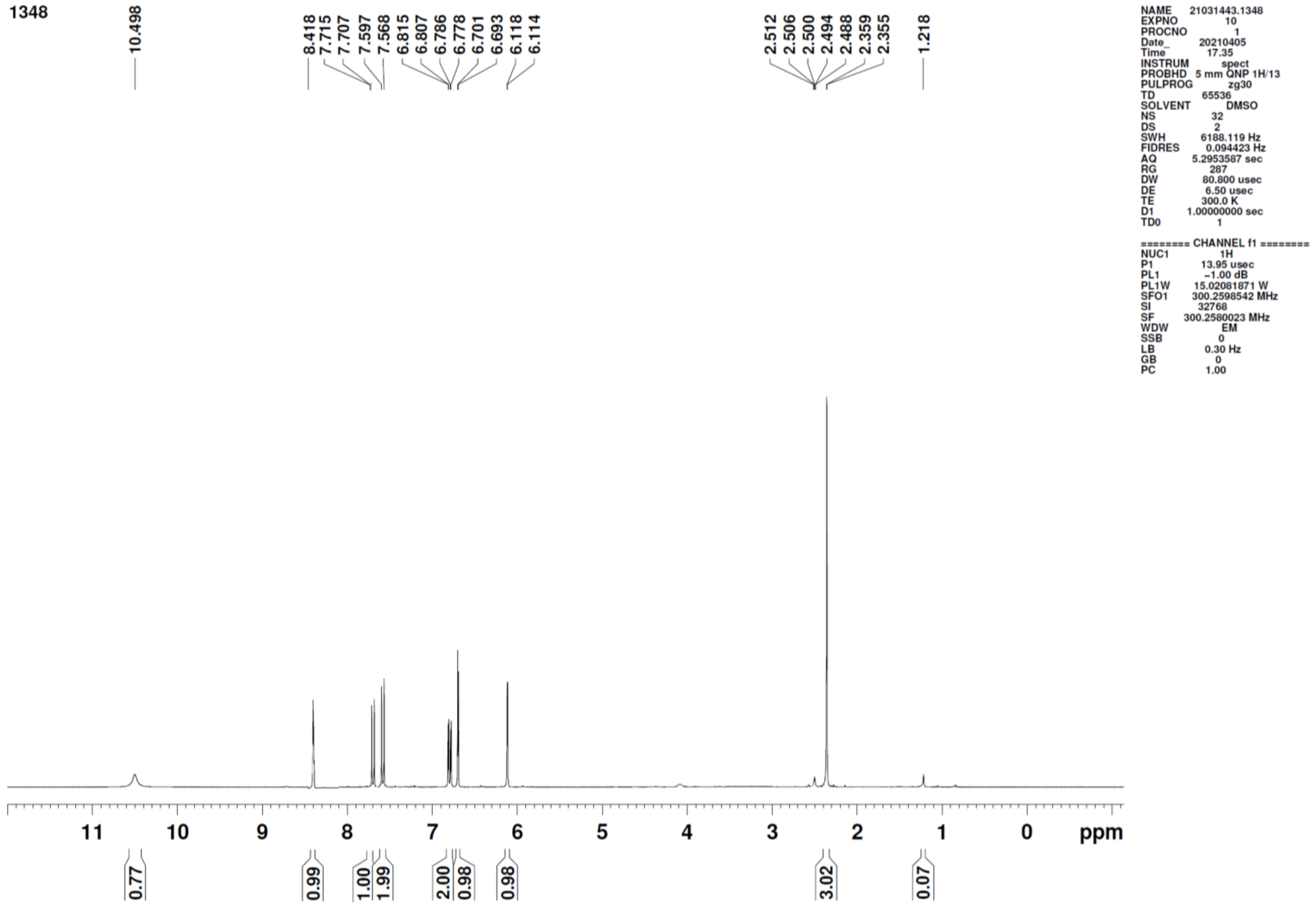

Spectra of *N*-(3-Chloro-4-fluorophenyl)-7-hydroxy-4-methyl-2-oxoquinoline-1(2*H*)-carboxamide (**5j**)

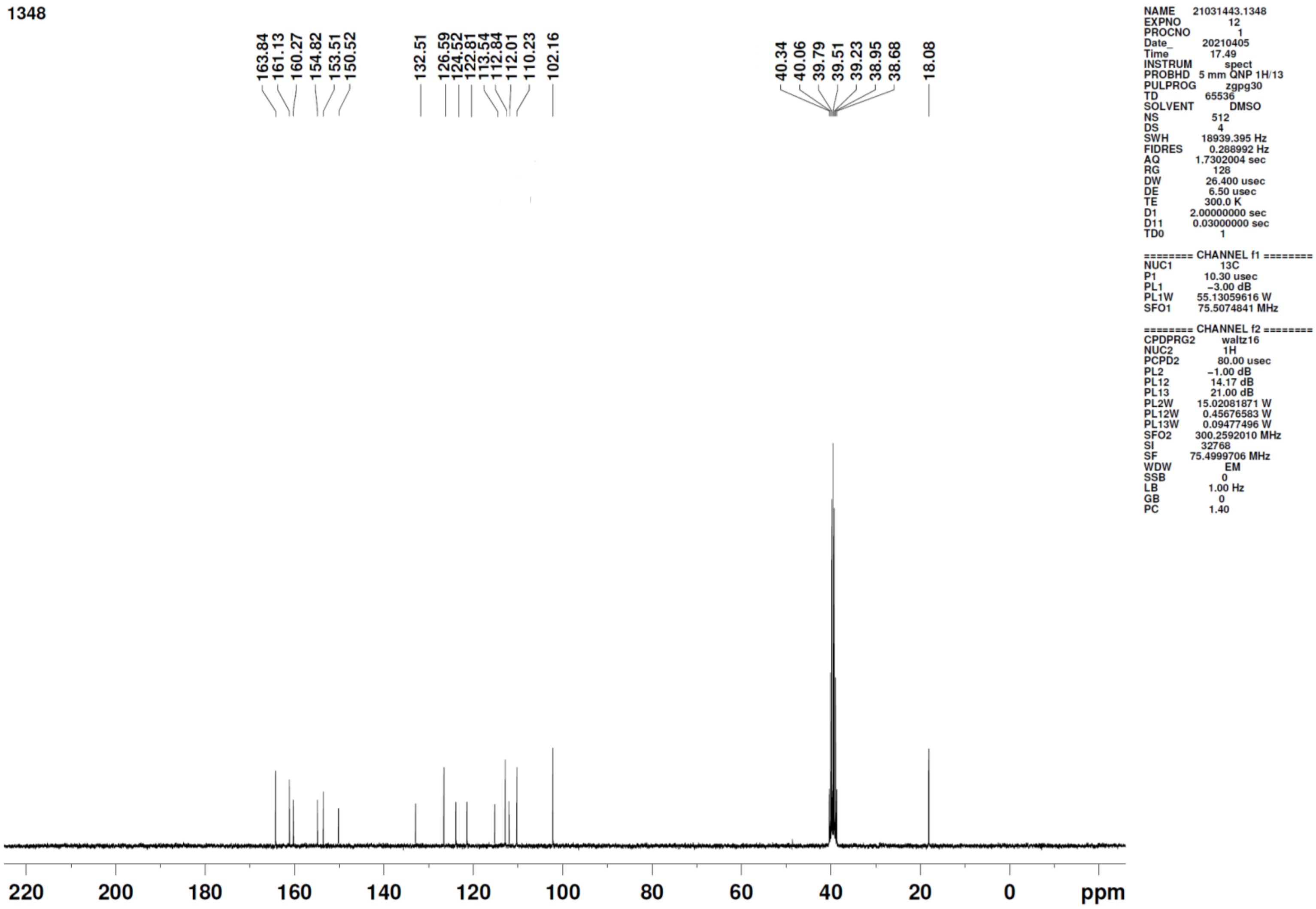

Spectra of *N*-(3-Chloro-4-fluorophenyl)-7-hydroxy-4-methyl-2-oxoquinoline-1(2*H*)-carboxamide (**5j**)
